# Geochronology, geochemistry, and geological significance of early Jurassic intrusive rocks in the Lesser Xing’an- Zhangguangcai Range, northeast China

**DOI:** 10.1371/journal.pone.0306465

**Published:** 2024-08-23

**Authors:** Zhonghai Zhao, Zhongju Li, Haina Li, Binbin Cheng, Yechang Yin

**Affiliations:** 1 College of Mining, Liaoning Technical University, Fuxin, Liaoning, China; 2 Liaoning Key Laboratory of Green Development of Mineral Resources, LNTU, Fuxin, Liaoning, China; 3 Northeast Geological S & T Innovation Center of China Geological Survey, Shenyang, Liaoning, China; 4 Shandong No.3 Exploration Institute of Geology and Mineral Resources, Yantai, Shandong, China; Tohoku University, JAPAN

## Abstract

The Lesser Xing’an—Zhangguangcai Range of northeast China is located in the eastern segment of the Central Asian Orogenic Belt (CAOB), which records intense magmatism during the Mesozoic. The petrogenesis and geodynamic setting of the Early Jurassic intrusive rocks in this region are unclear. In this paper, we present new zircon U–Pb age and whole-rock geochemical data for these intrusive rocks to investigate their origins and tectonic setting. Zircon U–Pb dating suggests these intrusive rocks were emplaced during the Early Jurassic (197–187 Ma). The granites are enriched in silica and alkali, and depleted in MgO and CaO. They are metaluminous to weakly peraluminous, and have high A/CNK values and low zircon saturation temperatures (T_Zr_ ~ 779°C), suggesting they are highly fractionated I-type granites derived by partial melting of lower crustal materials. The granites exhibit negative Nb, Ta, P, Eu, and Ti anomalies due to fractional crystallization. The diorites and gabbros have low SiO_2_ contents and high Mg^#^ values, and are enriched in light rare earth and large-ion lithophile (Ba, K, and Sr) elements, and depleted in heavy rare earth and high field strength (Nb, Ta, and Ti) elements. The geochemical characteristics show that the mafic magmas were derived by partial melting of mantle that had been metasomatized by subduction-related fluids. Based on the geochemical characteristics of coeval intrusive rocks and the regional geological setting, we suggest the Early Jurassic intrusive rocks in the Lesser Xing’an—Zhangguangcai Range were formed along an active continental margin, possibly as a result of bidirectional subduction of the Mudanjiang Oceanic plate between the Jiamusi and Songnen—Zhangguangcai Range massifs.

## Introduction

The Lesser Xing’an—Zhangguangcai Range in northeast (NE) China is located between the Jiamusi and Songnen massifs, and contains abundant Late Paleozoic to Mesozoic igneous rocks. It is a complex geological area that has experienced numerous tectonomagmatic events. Previous studies have reported extensive geochronological and geochemical data for plutons in this area, but the origin and tectonic setting of the igneous rocks are debated [1–3]. Some studies consider that delamination occurred beneath the Central Asian Orogenic Belt (CAOB), which led to the magma upwelling [4–6]; Whereas others have proposed that the igneous rocks were generated in a back-arc extensional setting associated with subduction of the Paleo-Pacific Plate [7–9]. Some recent studies have suggested that the igneous rocks are arc-related igneous rocks that formed during subduction in the Mudanjiang Ocean [10–14], which was located between the Jiamusi and Songnen massifs. Based on the arc-related intrusive rocks and active continental margin igneous rocks, subduction in the Mudanjiang Ocean is inferred to have begun the early Permian (>274 Ma) [12], Late Triassic [15, 16], or Early Jurassic [17]. The arc-related igneous rocks indicate the subduction polarity was bidirectional [12, 18] or westward [11.16].

Numerous igneous rocks occur in the Lesser Xing’an—Zhangguangcai Range, mainly granites and sparse intermediate—mafic intrusive rocks [14]. Therefore, previous studies of the intrusive rocks have focused mainly on the granites and their formation ages and petrogenesis. The granites were intruded during the Late Paleozoic and Mesozoic, particularly in the Late Triassic to Early Jurassic [10–12, 19–24]. Other studies have reviewed the spatiotemporal distribution of Late Paleozoic—Mesozoic granites in the Lesser Xing’an—Zhangguangcai Range, and noted that the granites exhibit a westward younging trend [25, 26]. The granites are mainly I- and A-type granites, and there are almost no S-type granite [10–12, 19–30]. Ge et al.(2020b) suggested the granitic magmas were derived by partial melting of Meso—Neoproterozoic crustal material [26]. Other studies have proposed that the granites were generated by partial melting of juvenile crust [19, 21, 24]. The granites might also were formed by the mixing of mantle- and crust-derived magmas [23].

Given that granites can form in a range of tectonic settings, studies of granite cannot uniquely constrain the tectonic evolution of the Lesser Xing’an—Zhangguangcai Range. Mafic igneous rocks are equally important for constraining the tectonic setting and magma sources. The most recent studies indicates the mafic rocks formed in the Early Jurassic [9, 25, 31]. Zircon Hf isotopes and geochemistry of igneous rocks indicate the mafic magmas were derived by partial melting of depleted mantle that had been metasomatized by subduction-derived fluids [9, 25, 31]. Therefore, we investigated the Early Jurassic intrusive rocks in the Shuguang Forest Farm area in the Lesser Xing’an—Zhangguangcai Range. We used petrographic, geochemical, and zircon U–Pb age data to determine the petrogenesis and tectonic setting of the intrusive rocks, which provide new insights into the tectonic evolution of the Lesser Xing’an—Zhangguangcai Range.

## Geological background

**Fig 1:**
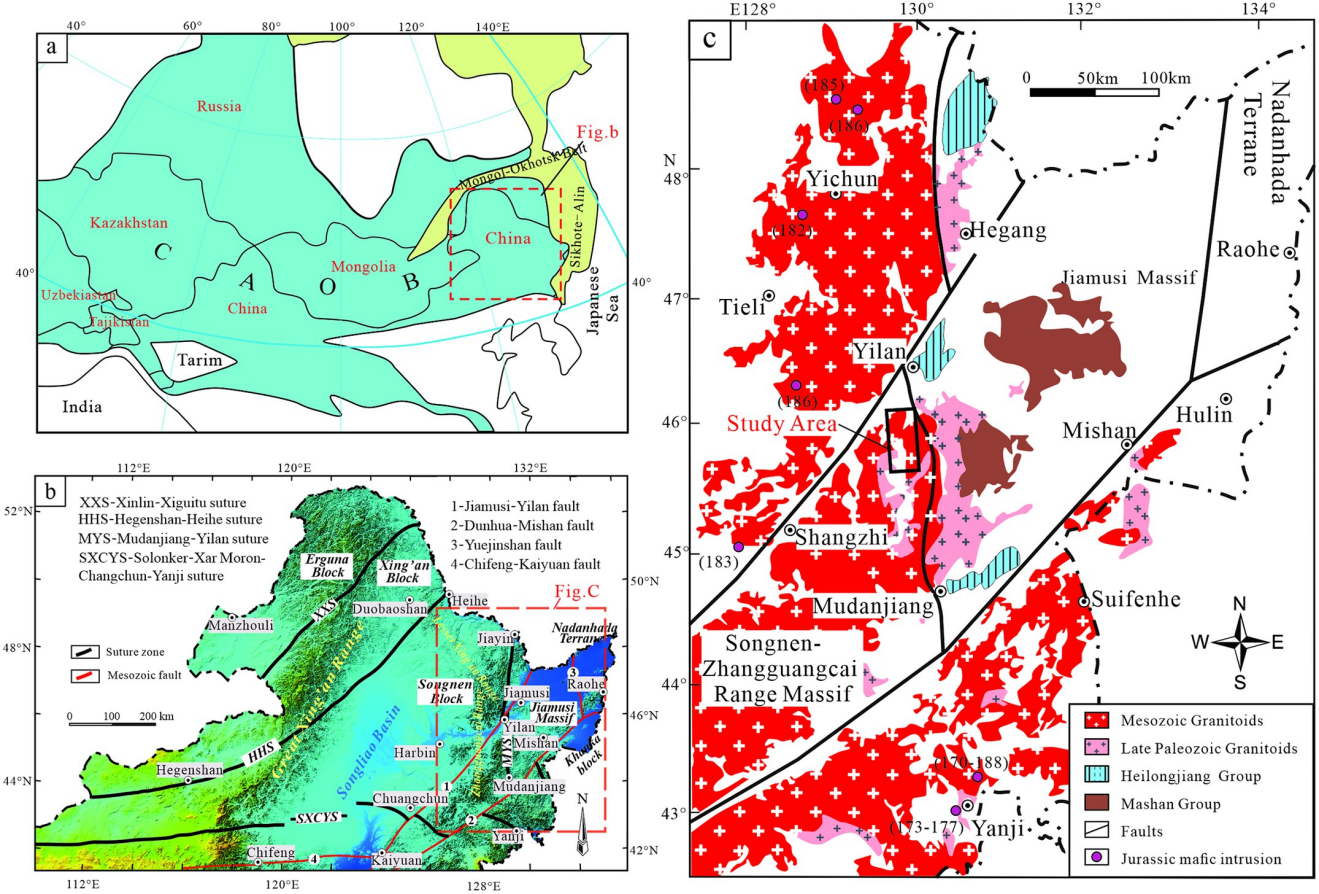
(a) Regional map showing the location of Central Asian Orogenic Belt; (b) Tectonic divisions sketch map of NE China; (c) Distribution of magmatic rocks in the Lesser Xing’an-Zhangguangcai Range. The original basemap was obtained from Natural Earth (http://www.naturalearthdata.com/) and was further processed using software ArcGIS 10.8 version. Digital elevation model was from the USGS National Map Viewer (http://viewer.nationalmap.gov/viewer/).

NE China is located between the North China Plate and the Siberian Plate, in the easternmost CAOB (Fig 1a [32]). It formed by complex tectonic and magmatic processes involving micro-continental plates, including the Ergun, Xing’an, Songliao, and Jiamusi massifs, and Nadanhada Terrane (from west to east) [14, 27, 32–35] (Fig 1b [14]). The study area is located in the Lesser Xing’an—Zhangguangcai Range, near the suture zone between the Jiamusi and Songnen—Zhangguangcai Range massifs, with the Jiamusi and Jiayin—Mudanjiang massifs to the east and Songnen Massif to the west.

The strata in the study area comprise the Lower Permian—Lower Triassic Hongguang Formation of the Zhangguangcai Range Group and Cenozoic—Quaternary rocks. The Hongguang Formation is distributed in a NE–SW to nearly N–S direction, and was intruded by Early Jurassic syenogranites, monzogranites, and granodiorites. The Hongguang Formation consists mainly of gray—black migmatites and intermediate—acidic volcanic rocks intercalated with marbles. The Quaternary strata are mostly distributed in linear bands along terraces and valleys on both sides of major rivers, and consist of clay and unconsolidated gravel.

The geological setting of the study area is complex and includes Late Archean—Mesoproterozoic continental breakup, Late Proterozoic—Early Paleozoic oceanic basin formation, Early Paleozoic convergence of the North China and Siberian plates (and other microplates) [36, 37], Late Paleozoic subduction and closure of the Paleo-Asian Ocean, collision of the southern margin of the Siberian Plate and northern margin of the North China Plate [38, 39], and westward subduction of the Pacific Plate and evolution of the Mongol—Okhotsk tectonic domain [19, 20, 40]. The study area contains abundant igneous rocks in a N–S-trending and sporadic upper Paleozoic strata [41] (Fig 1c [10, 14]). These igneous rocks are diverse and include acid, intermediate, and (ultra)mafic rocks, with granites being particularly common.

## Petrography

**Fig 2:**
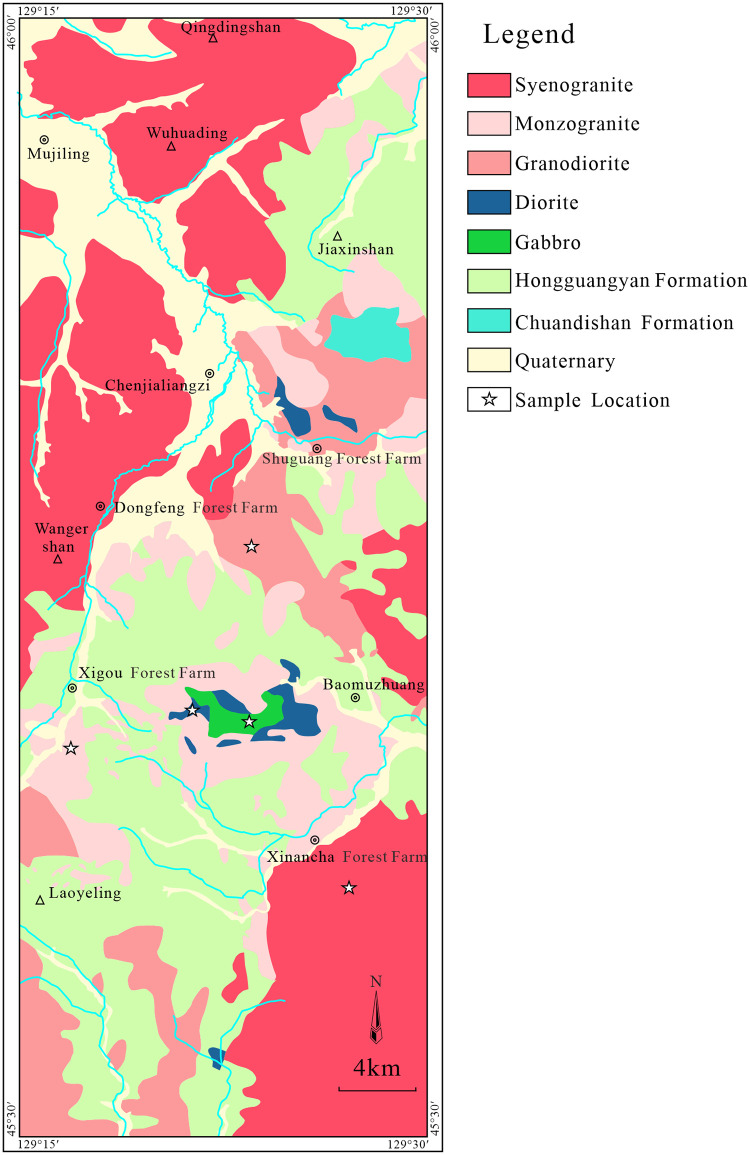
Simplified geological map of eastern Lesser Xing’an-Zhangguangcai Range showing sample locations. Image created by the authors in MapGIS6.7 and Core- lDRAW2020; no copyrighted material was used.

Early Jurassic intrusive rocks are widely distributed in the study area. Acid intrusive rocks are common and intermediate—mafic intrusive rocks are sporadically exposed, including syenogranite, monzogranite, diorite, and gabbro (Fig 2). To constrain the tectonic evolution of the Lesser Xing’an—Zhangguangcai Range, we sampled syenogranites, monzogranites, granodiorites, diorites, and gabbros.

**Fig 3:**
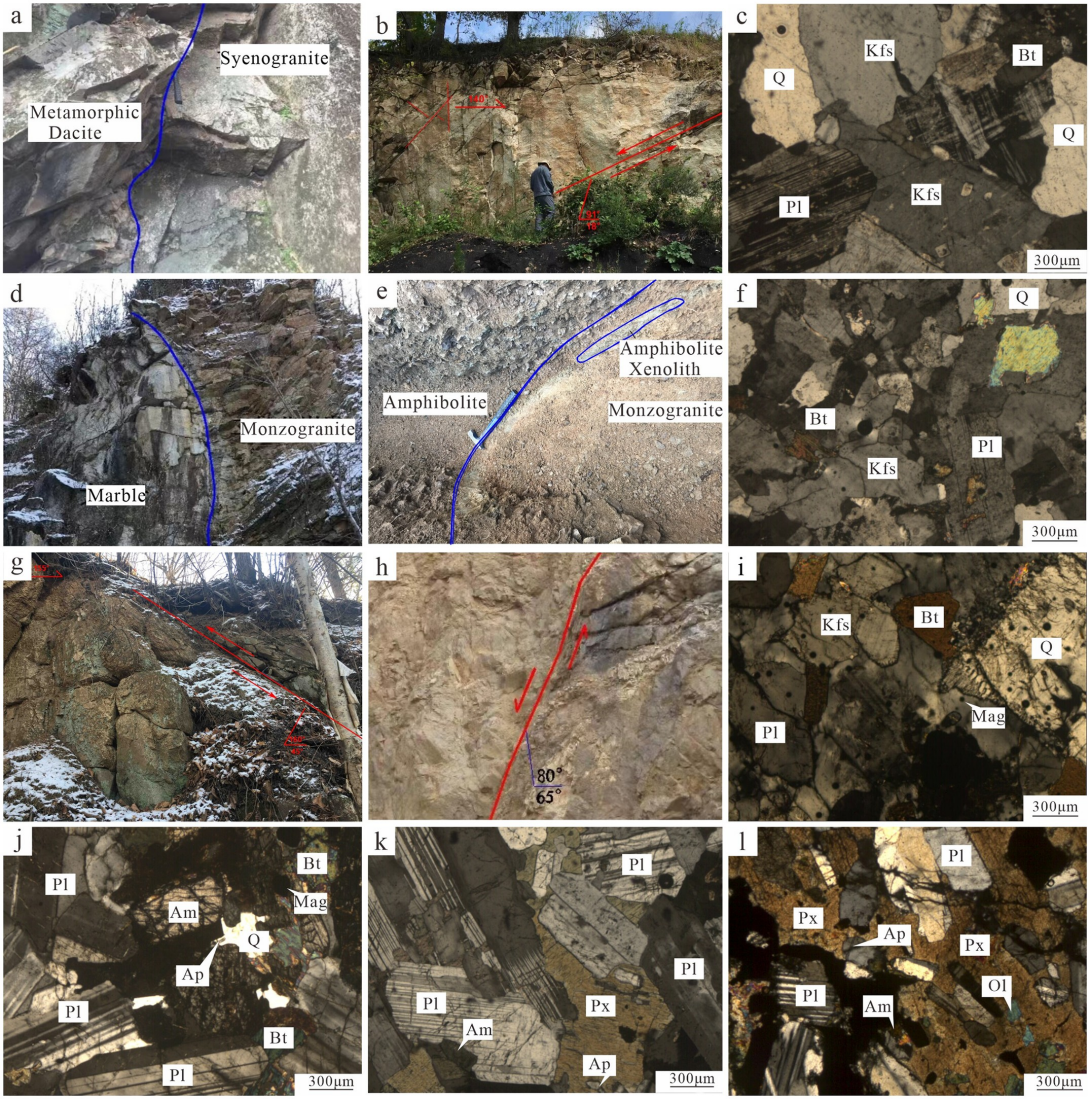
Field photographs and photomicrographs of intrusive rocks in the Shuguang forest farm. (a-c) Field photographs and photomicrographs of syenogranite; (d-f) Field photographs and photomicrographs of monzogranite; (g-i) Field photographs and photo- micrographs of granodiorite; (j-k) Microphotographs of the diorite; (l) Microphoto- graphs of the gabbro (Bt: biotite; Kfs: K-feldspar; Pl: plagioclase; Q: quartz; Am: amphibole; Ol: oivine; Px: pyroxene; Ap: apatite; Mag: magnetite).

The granitoid samples include syenogranite (TW01), monzogranite (TW02), and granodiorite (TW03). The plutons form batholiths and stocks. The Early Jurassic syenogranite intruded the Hongguangyan Formation and Early Jurassic granodiorite and monzogranite. The syenogranite is mainly fine- to medium-grained (Fig 3a–3c). The sample is red in color, with a fine-grained granitic texture and massive structure. It consists mainly of K-feldspar (47 vol.%), quartz (29 vol.%), plagioclase (22 vol.%), and biotite (2 vol.%). The monzogranite has a fine- to medium-grained granitic texture and massive structure (Fig 3d–3f), and consists of quartz (35 vol.%), K-feldspar (32 vol.%), plagioclase (25 vol.%), and biotite (8 vol.%). The granodiorite has a fine- to medium-grained granitic texture and massive structure (Fig 3g–3i), and consists of plagioclase (50 vol.%), quartz (25 vol.%), K-feldspar (15 vol.%), and biotite (10 vol.%). Accessory minerals include magnetite, zircon, titanite, and apatite.

The diorite (TW04) occurs in the form of a stock. The Early Jurassic diorite intruded a gabbro, and was intruded in turn by granodiorite and monzogranite. The sample is gray—black in color, with a fine-grained granular texture and massive structure (Fig 3j). It consists of plagioclase (70 vol.%), amphibole (20 vol.%), biotite (7 vol.%), and quartz (3 vol.%), with accessory magnetite and zircon.

The gabbro (TW05) occurs as a small stock, and is intruded by Early Jurassic diorite, granodiorite, and monzogranite. The sample is gray—black in color and has a gabbroic texture and massive structure (Fig 3k and 3l). The sample consists of pyroxene (40 vol.%), plagioclase (40 vol.%), amphibole (15 vol.%), olivine (5 vol.%), and accessory minerals are apatite, titanite, and zircon.

## Analytical methods

### Zircon U-Pb dating

The zircon grains were separated in the laboratory of the Langfang Fengzeyuan Rock and Ore Detection Technology Company, China. After mechanical crushing, and heavy liquid and magnetic separation, we handpicked relatively complete and transparent zircon grains under a binocular microscope. Zircons were mounted in epoxy resin and polished to expose their centres for cathodoluminescence (CL) imaging at the Beijing Createch Testing Technology Company, China. The zircons were dated by laser ablation—inductively coupled plasma—mass spectrometry (LA–ICP–MS). The data processing was undertaken with ICP–MS Data Cal software. The standard zircon 91500 was used as the external standard, and the standard zircon GJ-1 was used for monitoring data quality. NIST 610 were used as an external standard to calibrate zircon trace element contents, and ^29^Si was used as an internal standard. Common Pb was corrected following Andersen (2002) [42]. The U–Pb age calculations and concordia plots were obtained with Isoplot 3.0. The concordia ages are quoted at the 95% confidence level and the dating results are presented in Table 1.

**Table 1:** LA-ICP-MS zircon U-Pb dating results of intrusive rocks in the study area.

Sample	w_B_/10^−6^		Th/U	isotope ratio						U-Pb age / Ma					
	Th	U		^206^Pb/^238^U	1σ	^207^Pb/^235^U	1σ	^207^Pb/^206^Pb	1σ	^206^Pb/^238^U	1σ	^207^Pb/^235^U	1σ	^207^Pb/^206^P	1σ
TW01-01	248	777	0.32	0.031	0.0013	0.2122	0.0106	0.0493	0.0009	197	8	195	9	161	47
TW01-02	166	441	0.38	0.0306	0.001	0.2365	0.0119	0.0552	0.0014	194	6	216	10	420	56
TW01-03	32.3	299	0.11	0.0302	0.0006	0.2053	0.0079	0.0492	0.0014	192	4	190	7	167	67
TW01-04	403	1112	0.36	0.0258	0.0006	0.2035	0.0116	0.0577	0.0035	164	4	188	10	517	133
TW01-05	183	519	0.35	0.0298	0.0007	0.1928	0.006	0.0469	0.0011	189	4	179	5	43	59
TW01-06	95.9	493	0.19	0.0309	0.0008	0.2213	0.0062	0.0519	0.0008	196	5	203	5	280	37
TW01-07	497	1158	0.43	0.0302	0.0009	0.2125	0.0071	0.0509	0.0006	192	6	196	6	235	30
TW01-08	388	1256	0.31	0.0309	0.001	0.2168	0.0083	0.0508	0.0008	196	6	199	7	228	35
TW01-09	270	906	0.3	0.0298	0.0008	0.2138	0.0064	0.0523	0.0017	189	5	197	5	298	76
TW01-10	1325	2455	0.54	0.03	0.0011	0.2063	0.0078	0.0499	0.0009	190	7	190	7	191	44
TW01-11	534	1327	0.4	0.0305	0.0008	0.2083	0.0057	0.0496	0.0008	194	5	192	5	176	37
TW01-12	322	1064	0.3	0.0304	0.0012	0.2121	0.0078	0.0506	0.0005	193	8	195	7	233	22
TW01-13	150	627	0.24	0.0296	0.0006	0.223	0.016	0.0545	0.0038	188	4	204	13	394	156
TW01-14	257	949	0.27	0.0309	0.0008	0.2165	0.0076	0.0509	0.0013	196	5	199	6	235	62
TW01-15	265	905	0.29	0.0307	0.001	0.2152	0.0092	0.0517	0.0023	195	6	198	8	276	100
TW01-16	412	985	0.42	0.0305	0.0012	0.2522	0.0078	0.0616	0.0035	194	8	228	10	659	123
TW01-17	342	1017	0.34	0.0309	0.0009	0.2177	0.0123	0.051	0.001	194	8	228	6	123	123
TW01-18	422	1229	0.34	0.0308	0.0014	0.2075	0.0076	0.0488	0.0016	196	6	200	10	46	46
TW01-19	376	1045	0.4	0.0301	0.0007	0.2172	0.0108	0.0522	0.0009	196	9	191	6	76	76
TW01-20	126	629	0.44	0.0302	0.0012	0.2217	0.0078	0.0528	0.0009	191	4	200	10	39	39
TW02-01	485	477	1.02	0.0288	0.0005	0.1972	0.0064	0.0496	0.0015	183	3	183	5	176	75
TW02-02	162	173	0.94	0.029	0.0005	0.1965	0.0112	0.0493	0.003	184	3	182	9	165	141
TW02-03	173	286	0.6	0.0291	0.0009	0.1933	0.0105	0.0483	0.0025	185	6	179	9	122	110
TW02-04	198	234	0.85	0.0294	0.0004	0.1973	0.0064	0.0487	0.0015	187	3	183	5	132	79
TW02-05	213	292	0.73	0.0289	0.0006	0.2199	0.0125	0.0556	0.0033	183	4	202	10	435	135
TW02-06	135	170	0.79	0.0289	0.0006	0.2006	0.0088	0.0502	0.0021	184	4	186	7	206	96
TW02-07	658	507	1.3	0.0293	0.0006	0.2091	0.0139	0.0513	0.003	186	4	193	12	254	130
TW02-08	136	199	0.68	0.029	0.0005	0.211	0.0075	0.0526	0.0017	184	3	194	6	322	74
Sample	w_B_/10^−6^		Th/U	isotope ratio						U-Pb age / Ma					
	Th	U		^206^Pb/^238^U	1σ	^207^Pb/^235^U	1σ	^207^Pb/^206^Pb	1σ	^206^Pb/^238^U	1σ	^207^Pb/^235^U	1σ	^207^Pb/^206^P	1σ
TW02-09	174	230	0.76	0.0303	0.0006	0.2517	0.0137	0.0603	0.0031	192	4	228	11	613	111
TW02-10	631	350	1.8	0.0298	0.0006	0.21	0.0097	0.051	0.0022	189	4	194	8	239	72
TW02-11	275	252	1.09	0.029	0.0007	0.2092	0.009	0.0527	0.0026	184	4	193	8	317	111
TW02-12	73.5	174	0.42	0.0291	0.0005	0.2145	0.011	0.0532	0.0026	185	3	197	9	339	111
TW02-13	217	305	0.71	0.0295	0.0006	0.2255	0.0087	0.0557	0.0024	188	4	207	7	443	127
TW02-14	467	906	0.52	0.0295	0.0005	0.1904	0.006	0.0472	0.0016	187	3	177	5	58	143
TW02-15	268	309	0.87	0.0295	0.0006	0.206	0.0073	0.0506	0.0015	187	4	190	6	220	75
TW02-16	269	281	0.96	0.0309	0.0006	0.2126	0.0095	0.0499	0.0021	196	4	196	8	191	98
TW02-17	354	277	1.28	0.0297	0.0006	0.2233	0.0092	0.0541	0.0017	189	4	205	8	376	77
TW02-18	159	286	0.56	0.0295	0.0005	0.2012	0.008	0.0495	0.0019	187	3	186	7	169	89
TW02-19	222	309	0.72	0.0304	0.0005	0.2103	0.007	0.0502	0.0017	193	3	194	6	211	78
TW02-20	173	286	0.6	0.0303	0.0006	0.2078	0.0084	0.0499	0.0019	193	4	192	7	187	90
TW03-01	183	335	0.55	0.0309	0.0011	0.214	0.0081	0.0507	0.0011	196	7	197	7	228	54
TW03-02	96.6	201	0.48	0.0304	0.0013	0.2016	0.0091	0.0483	0.0013	193	8	186	8	122	61
TW03-03	74.8	154	0.49	0.0307	0.0011	0.2753	0.0255	0.0622	0.0033	195	7	247	20	683	114
TW03-04	102	194	0.53	0.0311	0.0011	0.2262	0.0101	0.0526	0.0009	197	7	207	8	309	8
TW03-05	92.6	175	0.53	0.0305	0.0008	0.2054	0.007	0.049	0.0013	194	5	190	6	150	61
TW03-06	97.5	164	0.59	0.0307	0.0008	0.2232	0.0081	0.0526	0.0012	195	5	205	7	322	52
TW03-07	143	261	0.55	0.0307	0.0008	0.2039	0.0057	0.0484	0.0008	195	5	188	5	120	44
TW03-08	70.4	171	0.41	0.0299	0.0015	0.226	0.0154	0.0537	0.0013	190	9	207	13	361	54
TW01-01	248	777	0.32	0.031	0.0013	0.2122	0.0106	0.0493	0.0009	197	8	195	9	161	47
TW03-09	275	415	0.66	0.0304	0.0014	0.2114	0.0108	0.051	0.0021	193	9	195	9	239	101
TW03-10	43.9	90.6	0.48	0.0303	0.0009	0.2076	0.0096	0.0497	0.0018	193	6	192	8	189	83
TW03-11	85.3	156	0.55	0.0307	0.001	0.2234	0.0225	0.0524	0.0041	195	6	205	19	306	177
TW03-12	190	249	0.76	0.0323	0.0012	0.2283	0.0098	0.0511	0.0011	205	7	209	8	256	51
TW03-13	81.4	170	0.48	0.0307	0.0012	0.2287	0.0124	0.0534	0.0013	195	7	209	10	346	90
TW03-14	104	188	0.55	0.0308	0.0013	0.2173	0.0114	0.0511	0.0014	195	8	200	10	256	63
TW03-15	337	586	0.58	0.0306	0.0008	0.2128	0.0065	0.0504	0.0008	194	5	196	5	217	32
Sample	w_B_/10^−6^		Th/U	isotope ratio						U-Pb age / Ma					
	Th	U		^206^Pb/^238^U	1σ	^207^Pb/^235^U	1σ	^207^Pb/^206^Pb	1σ	^206^Pb/^238^U	1σ	^207^Pb/^235^U	1σ	^207^Pb/^206^P	1σ
TW03-16	83.6	210	0.4	0.0309	0.0013	0.2172	0.0103	0.0509	0.001	196	8	200	9	235	43
TW03-17	143	275	0.52	0.0304	0.0012	0.2133	0.0103	0.051	0.0013	193	8	196	9	239	53
TW03-18	200	315	0.63	0.0305	0.0009	0.2105	0.0067	0.0503	0.0014	194	5	194	6	209	95
TW03-19	129	241	0.54	0.0307	0.0015	0.2168	0.0123	0.0511	0.0014	195	9	199	10	243	58
TW03-20	165	324	0.51	0.0304	0.0008	0.2168	0.0062	0.052	0.001	193	5	199	5	283	44
TW04-01	157	269	0.58	0.0307	0.0007	0.209	0.0063	0.0494	0.0009	195	5	193	5	169	41
TW04-02	173	233	0.74	0.031	0.0012	0.2206	0.0109	0.0512	0.0011	197	7	202	9	250	52
TW04-03	43.7	93.8	0.47	0.0309	0.0059	0.3028	0.0915	0.0728	0.0228	196	37	269	71	1009	673
TW04-04	183	237	0.77	0.031	0.0009	0.221	0.0079	0.0516	0.0009	197	6	203	7	265	36
TW04-05	40.7	104	0.39	0.0309	0.0006	0.1927	0.0196	0.0454	0.005	196	4	179	17	/	/
TW04-06	136	276	0.49	0.031	0.0007	0.2088	0.006	0.0488	0.0009	197	5	193	5	139	47
TW04-07	35.8	87.7	0.41	0.0308	0.0014	0.3064	0.0304	0.0697	0.0045	241	24	271	24	918	133
TW04-08	118	381	0.31	0.0381	0.0039	0.4899	0.0538	0.0932	0.0017	195	8	405	37	1494	35
TW04-09	49.9	227	0.22	0.0308	0.0013	0.2232	0.0084	0.0531	0.0011	194	7	205	7	345	51
TW04-10	70.3	218	0.32	0.0305	0.0012	0.2159	0.0079	0.0514	0.0011	198	8	199	7	261	48
TW04-11	31.5	73.3	0.43	0.0311	0.0013	0.2393	0.0144	0.0552	0.0015	199	13	218	12	420	56
TW04-12	118	202	0.58	0.0314	0.0021	0.2754	0.0214	0.0636	0.0028	196	6	247	17	728	94
TW04-13	139	201	0.69	0.031	0.0009	0.2165	0.0088	0.0506	0.0012	196	7	199	7	220	56
TW04-14	102	160	0.64	0.0309	0.0012	0.2268	0.0103	0.0532	0.0015	194	5	208	9	339	61
TW04-15	51.3	126	0.41	0.0306	0.0008	0.2221	0.0079	0.0525	0.0011	204	6	204	7	306	48
TW04-16	115	139	0.83	0.0321	0.0009	0.2126	0.0077	0.048	0.0011	241	24	196	6	102	49
TW04-17	89.7	163	0.55	0.0307	0.0009	0.2279	0.0102	0.0533	0.0012	195	5	208	8	343	47
TW04-18	331	377	0.88	0.0312	0.0012	0.2194	0.009	0.0509	0.0007	198	8	201	8	235	30
TW04-19	91.4	169	0.54	0.0315	0.002	0.2186	0.0178	0.0502	0.0028	200	12	201	15	211	133
TW04-20	282	317	0.89	0.0309	0.0008	0.2214	0.0072	0.0518	0.0007	196	5	203	6	276	33
TW05-01	23.8	61.6	0.39	0.031	0.001	0.2049	0.0092	0.048	0.0018	197	6	189	8	102	87
TW05-02	38	107	0.36	0.0305	0.0007	0.2108	0.009	0.0503	0.002	194	5	194	8	209	91
TW05-03	98.2	198	0.5	0.0305	0.0008	0.2057	0.0108	0.0489	0.002	193	5	190	9	139	94
Sample	w_B_/10^−6^		Th/U	isotope ratio						U-Pb age / Ma					
	Th	U		^206^Pb/^238^U	1σ	^207^Pb/^235^U	1σ	^207^Pb/^206^Pb	1σ	^206^Pb/^238^U	1σ	^207^Pb/^235^U	1σ	^207^Pb/^206^P	1σ
TW05-04	112	171	0.65	0.0307	0.0019	0.215	0.0155	0.0508	0.0015	195	12	198	13	232	69
TW05-05	54.6	192	0.28	0.0305	0.0009	0.2227	0.0104	0.0528	0.0015	194	6	204	9	317	67
TW05-06	88.4	292	0.3	0.0303	0.0015	0.2534	0.0135	0.0627	0.0041	192	9	229	11	698	139
TW05-07	127	244	0.52	0.0304	0.0008	0.212	0.0073	0.0507	0.001	193	5	195	6	233	72
TW05-08	120	202	0.59	0.0306	0.0014	0.2242	0.0134	0.0529	0.0016	194	9	205	11	324	69
TW05-09	22.6	57.6	0.39	0.0305	0.0033	0.2807	0.0355	0.0666	0.0044	194	21	251	28	828	141
TW05-10	14.5	53.7	0.27	0.0298	0.0007	0.2327	0.0124	0.0567	0.0028	189	4	212	10	480	107
TW05-11	514	680	0.76	0.0303	0.0009	0.1948	0.0064	0.0467	0.0008	193	6	181	5	32	41
TW05-12	39.5	125	0.32	0.0307	0.0008	0.2089	0.008	0.0493	0.0013	195	5	193	7	161	59
TW05-13	23.1	58.6	0.39	0.0311	0.001	0.2318	0.0102	0.0543	0.0019	197	6	212	8	383	78
TW05-14	23.1	58.1	0.4	0.0305	0.001	0.2344	0.0108	0.0567	0.0023	193	6	214	9	480	91
TW05-15	23.7	72.3	0.33	0.0309	0.0008	0.1851	0.0114	0.0433	0.0023	196	5	172	10	/	/
TW05-16	155	217	0.71	0.0307	0.001	0.2155	0.0075	0.0512	0.0012	195	6	198	6	256	54
TW05-17	40.8	80.8	0.5	0.0306	0.0008	0.2143	0.0073	0.0512	0.0017	194	5	197	6	256	74
TW05-18	38.3	130	0.29	0.0305	0.0012	0.2053	0.012	0.0492	0.0021	194	8	190	10	167	100
TW05-19	70.7	156	0.45	0.0306	0.001	0.2238	0.0086	0.0529	0.0011	195	6	205	7	324	48

### Whole-rock major and trace element analyses

We sampled representative intrusive rocks from outcrops and trenches in the study area for geochemical analysis. Major and trace element analysis was carried out at the Geological Research and Testing Center of First Geological Exploration institute of Heilongjiang Province, China. Fresh rock samples were washed with distilled water and air-dried. The samples were then dried in an oven at 120°C and powdered to 200 mesh with an agate mortar and pestle. Major and trace elements were determined by X-ray fluorescence (XRF) spectrometry with an accuracy of <3%. Trace elements were determined by ICP–MS. The major and trace element data are listed in Table 2.

**Table 2:** Composition of whole-rock major elements (wt.%) and trace elements (ppm) in the study area.

Sample	TW-01	TW-09	TW-11	TW-12	TW-02	TW-18	TW-19	TW-20	TW-03	TW-40	TW-65	TW-74
Lithology	Syenogranite				Monzogranite				Granodiorite			
SiO_2_	77.28	76.82	75.46	78.58	75.56	77.44	76.46	74.64	70.26	69.82	69.64	69.64
TiO_2_	0.11	0.1	0.04	0.1	0.17	0.1	0.08	0.19	0.38	0.41	0.34	0.39
Al_2_O_3_	12.12	13.15	13.36	11.47	12.19	11.31	12.16	12.3	14.92	14.66	15.21	15.37
Fe_2_O_3_	0.65	0.37	0.65	0.9	0.9	0.92	0.71	1.75	1.42	1.5	1.59	1.54
FeO	0.43	0.29	0.24	0.29	1.08	0.57	0.76	1.15	1.08	1.37	0.86	1.22
MnO	0.03	0.01	0.06	0.03	0.02	0.03	0.11	0.08	0.05	0.06	0.04	0.05
MgO	0.12	0.06	0.18	0.16	0.23	0.14	0.15	0.3	0.61	0.8	0.55	0.78
CaO	0.28	0.38	0.7	0.56	0.28	0.14	0.42	0.42	2.52	3.79	2.38	3.22
Na_2_O	3.2	3.13	3.49	3.38	2.98	3.2	3.79	4.22	4.23	4.21	4.45	4
K_2_O	4.75	5.28	4.96	4.52	5.57	5.26	4.36	4.43	3.26	2.17	3.1	2.66
P_2_O_5_	0.09	0.05	0.1	0.09	0.07	0.08	0.08	0.05	0.09	0.09	0.16	0.17
LOI	0.26	0.24	0.21	0.21	0.43	0.36	0.21	0.29	0.67	0.43	0.81	0.44
Total	99.32	99.88	99.45	100.29	99.48	99.55	99.29	99.82	100.2	99.83	99.84	99.87
A/NK	1.16	1.21	1.2	1.1	1.11	1.03	1.11	1.05	1.42	1.58	1.42	1.62
A/CNK	1.11	1.14	1.08	1	1.06	1.01	1.04	0.98	0.99	0.91	1.01	1
Mg^#^	17.41	14.65	28	20.59	17.83	15.15	16.05	16.41	31.56	34.4	29.97	34.79
La	48.58	12.1	21.38	35.43	44.6	23.66	95.06	43.58	33.02	36.26	28.55	26.27
Ce	88.56	26.25	37.79	56.31	102.61	42.18	85.7	99.08	67.02	72.08	50.76	45.17
Pr	8	3.01	4.64	6.43	9.83	5	16.61	8.62	7.33	7.51	6.69	5.96
Nd	24.93	10.3	16.18	20.88	32.09	17.62	55.37	28.87	27.64	26.91	23.86	21.08
Sm	3.42	2.17	3.17	3.47	5.19	4	7.6	4.25	4.42	3.91	4	3.62
Eu	0.48	0.21	0.19	0.28	0.22	0.49	0.3	1.4	1.23	1.25	1.23	1.33
Gd	3.45	1.86	2.66	3.16	4.4	3.55	6.55	4.08	3.97	3.82	3.5	3.26
Tb	0.49	0.43	0.49	0.47	0.65	0.73	0.82	0.6	0.57	0.53	0.51	0.49
Dy	2.86	3.4	3.49	2.63	3.82	4.99	3.8	3.57	2.93	2.6	2.58	2.45
Ho	0.55	0.76	0.81	0.49	0.75	1.02	0.64	0.73	0.52	0.46	0.46	0.45
Er	1.58	2.56	2.83	1.42	2.31	3.18	1.83	2.4	1.45	1.38	1.31	1.3
Tm	0.23	0.5	0.58	0.23	0.39	0.59	0.24	0.42	0.21	0.21	0.18	0.19
Yb	1.37	3.45	4.28	1.46	2.5	4.07	1.47	2.84	1.27	1.26	1.08	1.16
Lu	0.21	0.6	0.77	0.22	0.41	0.69	0.23	0.5	0.19	0.2	0.17	0.2
Y	14.53	22.42	24.02	14.36	23.77	31.93	16.79	20.6	14.37	12.98	15.55	15.66
Th	9.04	28.97	6.83	21.55	31.13	13.1	12.2	11.03	5.74	5.25	4.38	5.62
Nb	4.3	13.34	3.89	9.43	8.51	7.14	11.63	9.9	9.55	7.75	10.74	9.39
Sr	24.66	20.38	30.35	65.94	44.1	107.52	84.81	276.53	430.04	601.5	527.31	522.93
Ga	12.47	14.22	13.08	15.1	21.6	12.63	22.53	15.21	18.65	17.95	19.47	18.56
Ta	0.19	1.15	0.31	0.87	0.55	1.02	0.64	0.57	0.59	0.61	0.62	0.72
Zr	90.42	101.23	83.47	175.32	229.39	90.05	426.68	381.48	108.88	75.52	166.08	126.85
Ba	137.39	40.9	100.83	257.42	176.76	308.93	173.69	1425.38	983.23	1340.24	967.05	715.68
δEu	0.42	0.31	0.19	0.25	0.14	0.39	0.13	1.02	0.88	0.98	0.98	1.16
LREE	173.97	54.04	83.35	122.8	194.54	92.95	260.64	185.8	140.66	147.92	115.09	103.43
HREE	10.74	13.56	15.91	10.08	15.23	18.82	15.58	15.14	11.11	10.46	9.79	9.5
ΣREE	184.71	67.6	99.26	132.88	209.77	111.77	276.22	200.94	151.77	158.38	124.88	112.93
(La/Yb)N	23.91	2.36	3.37	16.36	12.03	3.92	43.6	10.35	17.53	19.4	17.82	15.27
Ce_N_/Yb_N_	17.95	2.11	2.45	10.71	11.4	2.88	29.42	9.69	14.66	15.89	13.05	10.82
T_Zr_	750	760	738	797	825	741	884	863	744	706	782	757
Sample	TW-04	TW-16	TW-17	TW-24	TW-05		TW-22		TW-23		TW-47	
Lithology	Diorite				Gabbro							
SiO_2_	60.58	57.36	56.96	54.62	49.86		48.81		47.48		49.26	
TiO_2_	0.84	0.94	0.89	0.68	2.6		1.95		1.55		1.2	
Al_2_O_3_	14.34	16.49	18.33	19.23	16.57		17.17		17.02		17.84	
Fe_2_O_3_	3.71	4.19	3.14	3.03	3.18		4.45		5.37		3.2	
FeO	3.16	3.74	3.95	3.81	3.59		4.64		5.14		4.17	
MnO	0.14	0.18	0.14	0.15	0.18		0.19		0.19		0.15	
MgO	2.99	3.67	2.76	3.25	5.89		6.78		6.3		6.36	
CaO	7.99	7.71	7.01	9.11	12.86		11.78		13.18		12.84	
Na_2_O	3.74	3.41	4.37	3.63	2.32		2.19		2.26		2.53	
K_2_O	2.02	1.57	1.71	1.08	0.8		1		0.41		0.46	
P_2_O_5_	0.08	0.07	0.09	0.07	0.08		0.07		0.05		0.1	
LOI	0.18	0.41	0.52	0.7	1.33		0.46		0.65		1.27	
Total	99.77	99.74	99.87	99.36	99.26		99.49		99.6		99.38	
A/NK	1.72	2.25	2.03	2.69	3.54		3.66		4.09		3.83	
A/CNK	0.63	0.77	0.84	0.81	0.59		0.66		0.6		0.64	
Mg^#^	45.06	46.55	42.07	46.99	61.94		58.3		52.97		61.66	
La	28.55	24.8	25.19	23.37	14.85		13.46		13.37		12.32	
Ce	53.76	49.16	48.3	48.39	29.41		27.75		31.51		31.29	
Pr	7.46	6.86	6.59	6.91	4.42		4.36		4.9		4.23	
Nd	29.91	28.11	26.64	27.28	19.77		20.49		21.91		17.15	
Sm	6.11	5.92	5.49	5.85	5.2		5		4.41		3.77	
Eu	1.7	1.66	1.68	1.36	1.7		1.9		1.56		1.23	
Gd	5.37	5.23	4.62	4.91	3.67		4.31		4.42		3.29	
Tb	1.01	0.98	0.88	0.97	0.73		0.88		0.9		0.64	
Dy	6.57	6.35	5.63	6.36	4.86		5.91		6.06		4.19	
Ho	1.29	1.24	1.11	1.26	0.94		1.17		1.2		0.81	
Er	3.69	3.59	3.17	3.62	2.64		3.2		3.37		2.25	
Tm	0.59	0.56	0.5	0.6	0.42		0.5		0.53		0.36	
Yb	3.66	3.42	2.99	3.69	2.5		3.05		3.21		2.1	
Lu	0.58	0.54	0.47	0.56	0.39		0.48		0.49		0.33	
Y	36.89	35.27	31.66	36.6	26.33		32.3		33.45		23.06	
Th	5.63	4.42	2.9	3.7	1.84		1.35		0.87		0.57	
Nb	8.12	5.41	7.62	10.31	4.99		4.7		3.87		1.7	
Sr	493.32	515.09	573.86	549.07	662.47		644.91		553.61		550.04	
Pb	12.6	10.1	8.1	9.9	4.4		4.8		3.2		3.4	
Co	18.76	20.96	22.6	18.9	54.72		52.3		37.12		56.56	
Cr	40.04	45.68	51.62	61.04	39.58		12.24		178.9		94.48	
Ni	14.36	15.81	11.18	8.07	14.65		7.55		27.6		23.51	
Ta	0.5	0.34	0.4	0.59	0.23		0.22		0.19		0.12	
Zr	493.32	515.09	573.86	549.07	662.47		644.91		553.61		550.04	
Ba	681.98	542.69	614.85	519.91	284.87		301.73		187.8		152.68	
δEu	0.89	0.89	0.99	0.76	1.26		1.22		0.97		1.05	
LREE	127.49	116.51	113.89	113.16	73.17		71.82		78.42		72.52	
HREE	22.76	21.91	19.37	21.97	16.15		19.5		20.18		13.97	
ΣREE	150.25	138.42	133.26	135.13	89.32		91.32		98.6		86.49	
(La/Yb)_N_	5.26	4.89	5.68	4	2.98		2.81		3.96		4	
Ce_N_/Yb_N_	4.08	3.99	4.49	3.64	3.27		2.53		2.73		4.14	

## Results

### Zircon U-Pb ages

**Fig 4:**
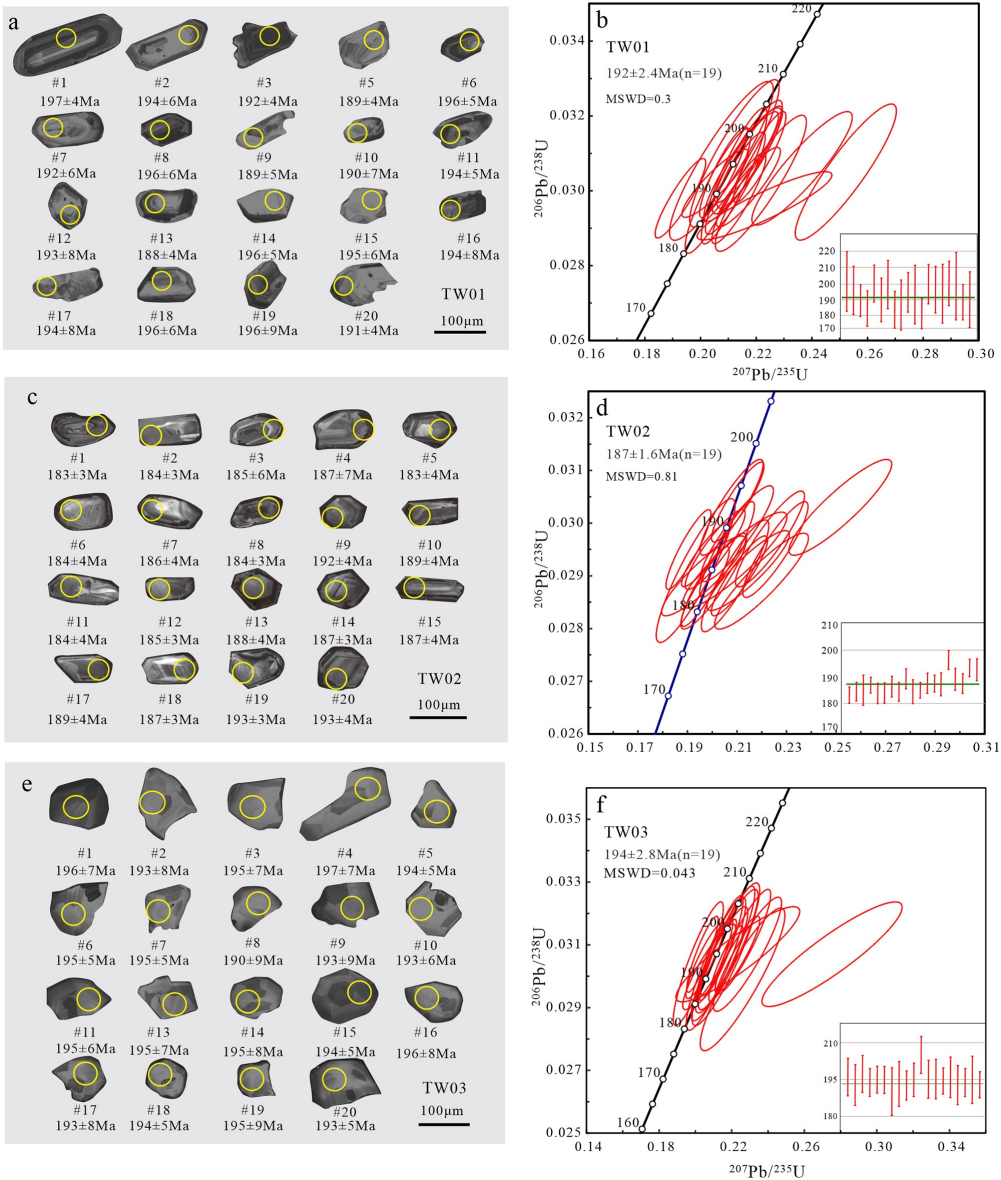
The CL images (a,c,e) and zircon U-Pb concordia diagrams (b,d,f) for granites.

**Fig 5:**
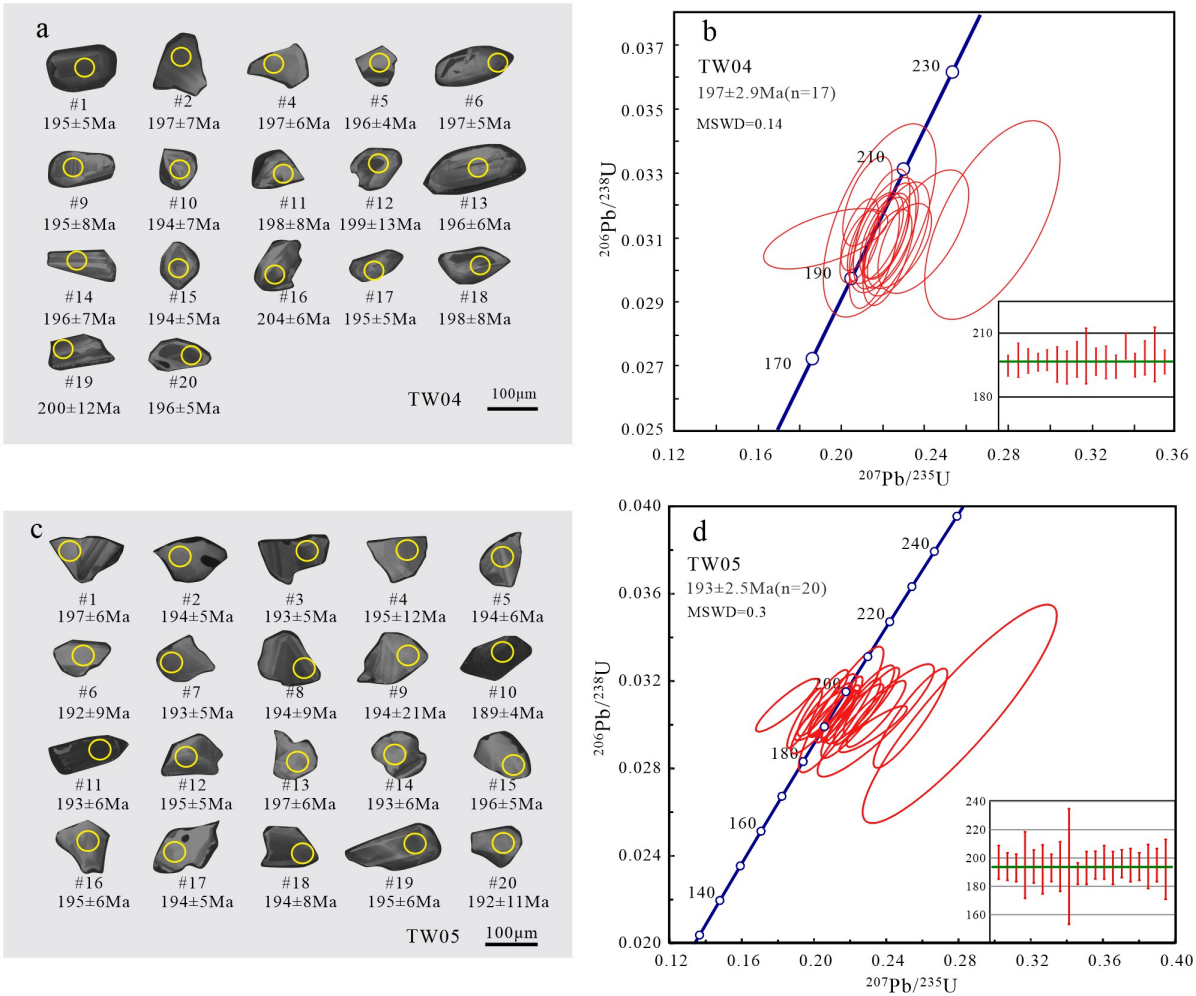
The CL images (a,c) and zircon U-Pb concordia diagrams (b,d) for diorite and gabbro.

Zircon grains from five intrusive rocks were selected for zircon U-Pb dating, including syenogranite, monzogranite, granodiorite, diorite and gabbro. The zircon crystals show short columnar, euhedral-subhedral in shape and bipyramidal development, with length:width ratios of 1:1~2:1. The grains have clear oscillatory zoning structure in the CL images (Figs 4a, 4c, 4e, 5a and 5c). All the zircon grains yield high Th/U ratios (0.11–1.80), indicating typical magmatic origin [38, 39].

The syenogranite was collected from the Yingzuilazi-Diaoshuihu pluton. A total of 20 zircon U-Pb ages were selected and tested, and one discordant age was eliminated. The ^206^Pb/^238^U ages given by 19 zircon grains range from 188 to 197 Ma, and yielded the weighted mean ^206^Pb/^238^U age of 192±2.4 Ma (MSWD = 0.3) (Fig 4b). This indicates the syenogranite was formed in Early Jurassic.

The monzogranite was sampled both sides of south-central Laoyeling highland and the northern part area around the Jiaxinshan-Weixing section. A total of 20 zircon U-Pb ages were selected and tested, excluding one discordant age. The ^206^Pb/^238^U ages given by 19 zircon grains range from 183 to 193 Ma, and yielded the weighted mean ^206^Pb/^238^U age of 187±1.6 Ma (MSWD = 0.81) (Fig 4d). This indicates that the formation age of monzogranite is Early Jurassic.

The granodiorite was collected from the southern pluton of Shuguang forest farm. The zircon grains are small, ranging from 90 to 100 μm, and most of the zircon grains are 90 μm. A total of 20 zircon U-Pb ages were selected and tested, and one discordant age was eliminated. The ^206^Pb/^238^U ages given by 19 zircon grains range from 190 to 197 Ma, and the weighted mean ^206^Pb/^238^U age was 194±2.8 Ma (MSWD = 0.043) (Fig 4f). This suggests that the formation age of granodiorite is Early Jurassic.

The diorite was taken from the 945 highland-698 highland pluton in the north of Laoyeling. The zircon grains are large, ranging from 100 to 130 μm. Most of the zircon grains are 100 μm, and the large ones can reach more than 150 μm. A total of 20 zircon U-Pb ages were selected and tested, and 3 discordant ages were removed. The ^206^Pb/^238^U ages given by 17 zircon grains range from 194 to 204 Ma, and yielded the weighted mean ^206^Pb/^238^U age of 197±2.9 Ma (MSWD = 0.14) (Fig 5b). It indicates that the diorite was formed in Early Jurassic.

The gabbros were collected from the 945 highland pluton in the north of Laoyeling. In the sample, the zircon grains are large, ranging from 100 to 150 μm. A total of 20 zircon U-Pb ages were selected and tested. The ^206^Pb/^238^U ages given by 20 analytical zircon grains range from 189 to 197 Ma, and yielded the weighted mean ^206^Pb/^238^U age of 193±2.5 Ma (MSWD = 0.3) (Fig 5d). It suggests that the gabbros were formed in Early Jurassic.

### Major elements

**Fig 6:**
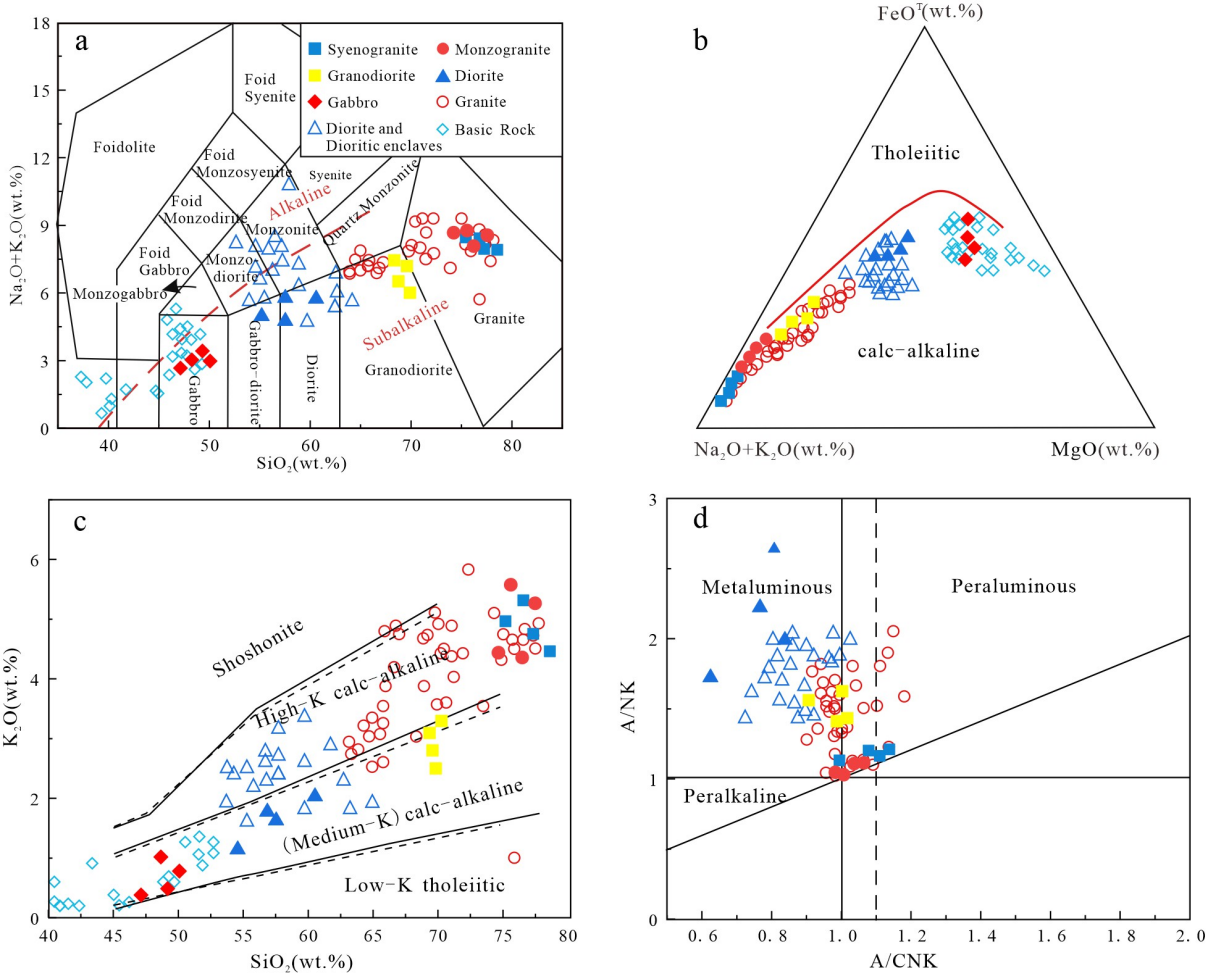
(a) TAS diagram of intrusive rocks (Base map is cited from [43]); (b) FeO^T^-(Na_2_O+K_2_O)-MgO diagram of intrusive rocks (Base map is cited from [44]); (c) K_2_O-SiO_2_ diagram (Base map is cited from [45]); (d) A/NK-A/CNK diagram of intrusive rocks (Base map is cited from [46]) (Basic rock data cited from [9, 25, 31], Granite data cited from [1, 22, 26, 31, 47–49], The data of diorite and dioritic enclaves are cited from [1, 9, 47, 48]. The date can be referred to S1 Table).

The SiO_2_ contents of the granites are 69.64~78.58 wt.%, the contents of total alkali (K_2_O+Na_2_O) are 6.38%~8.45 wt.%, and the contents of Al_2_O_3_ are 11.31~15.37 wt.%, with low MgO and P_2_O_5_ contents. All granitoid samples plot in the granite field in the TAS diagram (Fig 6a). These granites are calc-alkaline series in FeO^T^-(Na_2_O+K_2_O)-MgO diagram (Fig 6b). Granites are high-K calc-alkaline series in K_2_O-SiO_2_ diagram (Fig 6c). Their A/CNK ratios range from 1.00 to 1.14, belonging to the weakly peraluminous granites (Fig 6d).

The diorites contain SiO_2_ = 54.62~60.58 wt.%, Al_2_O_3_ = 14.34~19.23 wt.%, MgO = 2.76~3.67 wt.%, Mg^#^ = 42.07~46.99, and total alkalis contents (Na2O+K2O) = 4.71~6.08 wt.%. Diorite samples plot in the diorite area in the TAS diagram (Fig 6a). They are calc-alkaline series in FeO^T^-(Na_2_O+K_2_O)-MgO diagram (Fig 6b). They are medium-K calc-alkaline series in K_2_O-SiO_2_ diagram (Fig 6c). Their A/CNK ratios range from 0.67 to 0.71, showing metaluminous composition (Fig 6d).

The gabbros show compositional ranges with SiO_2_ = 47.48~49.86 wt.%, Al_2_O_3_ = 16.57~17.84 wt.%, K_2_O = 0.41~1.0 wt.%, MgO = 5.89~6.78 wt.%, Mg^#^ = 53.0~61.9, and total alkalis contents (Na2O+K2O) = 2.7~3.2 wt.%. Samples plot in the gabbroic area (Fig 6a). They are calc-alkaline series in FeO^T^-(Na_2_O+K_2_O)-MgO diagram (Fig 6b). They are medium-K and high-K calc-alkaline series in K_2_O-SiO_2_ diagram (Fig 6c).

### Rare earth elements and trace elements

**Fig 7:**
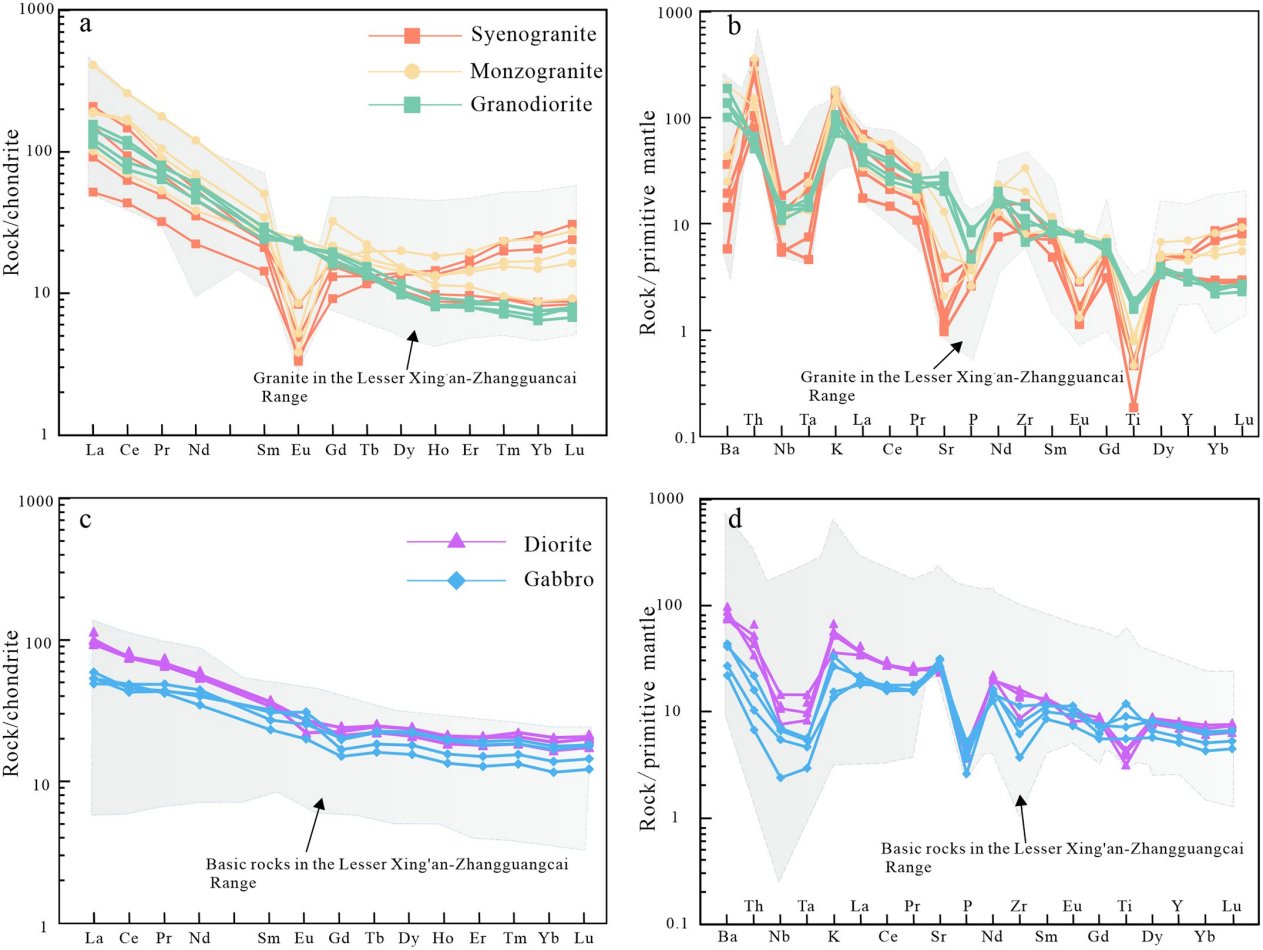
Chondrite-normalized REE patterns and primitive-mantle-normalized trace element diagrams of intrusive rocks in the study area (chondrite and primitive mantle values are from [50]) (the data source of the shadow part is consistent with Fig 6).

The syenogranite, monzogranite, granodiorite, diorite and gabbro all show a right-inclined curve of enrichment light rare earth element (LREE) and depletion heavy rare earth element (HREE) in the chondrite-normalized rare earth element (REE) diagram (Fig 7a and 7c). The syenogranite and monzogranite have obvious negative Eu anomalies. The syenogranite, monzogranite and granodiorite are characterized by enrichment of large-ion lithophile elements (LILE, e.g., Sr, K) and depletion of high field-strength elements (HFSE, e.g., Nb, P, Ti, Ta) in the primitive mantle-normalized spider diagram (Fig 7b). The diorites and gabbros are characterized by enrichment of large-ion lithophile elements (LILE, e.g., Ba, Sr, K) and depletion of high strength-field elements(HFSE, e.g., Nb, P, Ta, Zr) (Fig 7d).

## Discussion

### Ages of the intrusive rocks from the Lesser Xing’an- Zhangguangcai Range

In recent years, previous studies have carried out on magmatism, plate amalgamation, tectonic setting in the Lesser Xing’an-Zhangguangcai Range. Moreover, they have conducted extensive research on the formation age and petrogenetic types of rocks [9–12, 19–26, 28–30]. Some studies consider that the formation ages of the granites in the Lesser Xing’an-Zhangguangcai Range were mainly in the Mesozoic-Late Paleozoic, with the most intense magmatic activity occurring from Late Triassic to Early Jurassic [10–12, 19–24]. Only minor granites were formed in the Neoproterozoic and Early Paleozoic [13, 14, 29, 30]. Other studies have proposed that the mafic-ultramafic intrusive rocks exposed in Shuguang forest farm, Liuzhonggou, Xincun, Pingfang and Yichun areas of the Lesser Xing’an-Zhangguangcai Range and the diabase in Tieli area [9, 25, 31]. These studies show that the mafic-ultramafic rocks are formed in the Early Jurassic. In recent years, previous have carried out abundant zircon U-Pb dating studies on the pluton in the Lesser Xing’an-Zhangguangcai Range. And the Early Paleozoic pluton is corrected to late Paleozoic and Mesozoic [27, 51]. In this paper, we conducted zircon U-Pb dating on five types of intrusive rocks in the study area. The ages of intrusive rocks are 197–187 Ma. In summary, this is consistent with the previous geological study results.

**Fig 8:**
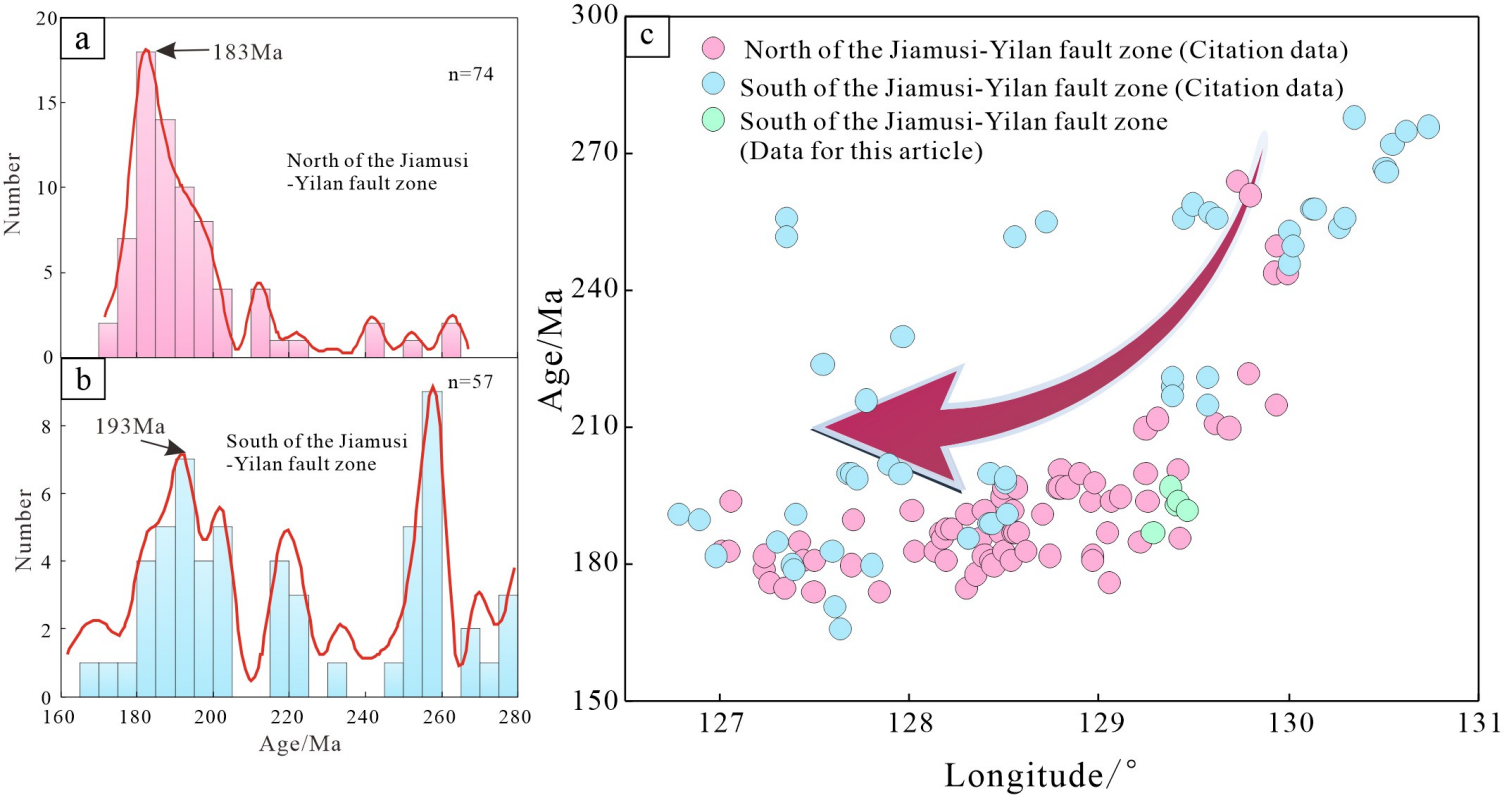
Zircon U-Pb ages characterization of intrusive rocks in the Lesser Xing’an- Zhangguangcai Range.

The spatiotemporal distribution pattern of intrusive rocks in the Lesser Xing’an-Zhangguangcai Range is of significance to explore the tectonic setting of the region. Ge et al.(2020b) collected formation ages of Late Paleozoic-Mesozoic granites, showing a spatiotemporal characteristic of gradually becoming juvenile from east to west [26]. In this paper, we not only collects the age of granites, but also adds the age of intermediate—mafic intrusive rocks (Table 3). Due to this area is located near the Jiamusi-Yilan fault of the northern section of the Tanlu fault. In order to eliminate the influence of strike-slip displacement caused by the Jiamusi-Yilan fault, the age data are divided into two groups based on the north and south sides of the fault to plot diagrams (Fig 8a–8c). We find that the Late Paleozoic-Mesozoic intrusive rocks gradually become juvenile from east to west.

**Table 3:** Age statistics of late paleozoic to mesozoic intrusive rocks in the Lesser Xing’an-Zhangguangcai Range.

Order	Sample	Location	latitude	longitude	Lithology	Method	Age	References
The north side of Jiamusi-Yilan fault								
1	SDQ1410	Sandaowanzi	50°22’13″N	127°00’52″E	Granodiorite	LAICP-MS	183 ± 2	Liu J Y,2016 [52]
2	SDQ1423	Sandaowanzi	50°21’23″N	127°03’03″E	Syenogranite	LAICP-MS	183 ± 1	Wang S S,2017 [53]
3	SDQ1425	Sandaowanzi	50°22’18″N	127°03’37″E	Syenogranite	LAICP-MS	194 ± 2	Wang S S,2017 [53]
4	H15-61-5	Yuquan	45°23’27″N	127°13’42″E	Syenogranite	LAICP-MS	179 ± 3	Ge M H,2017 [10]
5	DKW41	Wudaoling	45°18’13″N	127°13’52″E	Sericitolite	LA-ICPMS	179 ± 3	Tan H Y,2012 [54]
6	HWC1-1	Pingfangdian	45°08’29″N	127°14’02″E	Gabbrodiorite	LA-ICPMS	182 ± 2	Zhao L,2019 [55]
7	HSW2-6	Xingfu forest farm	48°42’51″N	127°15’43″E	Monzogranite	LAICP-MS	176 ± 1	Xu M J,2013 [19]
8	HSW2-3	Fengshuigouhequn	49°43’07″N	127°20’23″E	Monzogranite	LAICP-MS	175 ± 1	Xu M J,2013 [19]
9	HSW6-12	Xingfu forest farm	48°41’55″N	127°25’15″E	Syenogranite	LAICP-MS	185 ± 2	Xu M J,2013 [19]
10	DKG2	Gongpengzi	45°33’06″N	127°26’40″E	Granodiorite	LA-ICPMS	181± 2	Tan H Y,2012 [54]
11	DKY11	Yuanjiatun	45°26’43″N	127°29’37″E	Granodiorite	LA-ICPMS	174 ± 2	Tan H Y,2012 [54]
12	H15-63-4	maoershan	45°17’03″N	127°29’59″E	Monzogranite	LA-ICPMS	181 ± 1	Ge M H,2017 [10]
13	H15-66-1	jidiantun	45°23’19″N	127°41’12″E	Monzogranite	LA-ICPMS	180 ± 1	Ge M H,2017 [10]
14	DY0556-1	jidiantun	45°23’03″N	127°41’45″E	Syenogranite	LA-ICPMS	190 ± 1	Wu F Y,2011 [14]
15	H15-68-1	Yimancun	45°19’27″N	127°50’31″E	Syenogranite	LA-ICPMS	174 ± 1	Ge M H,2017 [10]
16	D5354	liuhetun	46°21’42″N	128°00’36″E	Hornblendite	LAICP-MS	192 ± 3	Li H N,2021 [47]
17	HYL1-1	Liuzhonggou	46°20’54″N	128°01’08″E	Hornblendite	LA-ICPMS	183 ± 1	Zhao L,2019 [55]
18	97SW005	Yimaianpo	46°05’05″N	128°07’32″E	Granodiorite	SHRIMP	183 ± 4	Wu F Y,2011 [14]
19	D6	liuhetun	46°26’23″N	128°09’18″E	Monzogranite	LAICP-MS	187±4	Li H N,2023 [47]
20	E6	liuhetun	46°27’31″N	128°10’11″E	Dioritic enclaves	LAICP-MS	186±2	Li H N,2023 [48]
21	H15-08-1	Taoshanzhen	46°55’05″N	128°11’06″E	Diorite	LAICP-MS	181 ± 1	Ge M H,2018 [11]
22	H15-09-1	Taoshanzhen	46°55’05″N	128°11’06″E	Monzogranite	LA-ICPMS	188 ± 1	Ge M H,2020(b) [26]
23	QF-1	liuhetun	46°25’35″N	128°13’15″E	Granodiorite	LAICP-MS	188±3	Li H N,2023 [48]
24	Jan-77	Shichang	46°56’17″N	128°17’51″E	Granodiorite	LA-ICPMS	175 ± 2	Wu F Y,2011 [14]
25	D2713	Pingdingshan	46°30’45″N	128°18’16″E	Syenogranite	LA-ICPMS	191±3	Yin Z G,2021 [22]
26	DKX12	Erguxishan	47°12’11″N	128°18’48″E	Granite	LA-ICPMS	186 ± 2	Tan H Y,2012 [54]
27	H15-11	Shenshuzhen	46°56’17″N	128°20’42″E	Granodiorite	LAICP-MS	178 ± 2	Ge M H,2018 [11]
28	DKE12	Ergu	47°11’25″N	128°22’07″E	Granodiorite	LA-ICPMS	186±2	Tan H Y,2012 [54]
29	PM07LT46	Tieli	46°47’41″N	128°23’12″E	Syenogranite	LA-ICPMS	192 ± 1	Zhen B,2016 [56]
30	HYC10-1	Xinghuo	47°42’37″N	128°23’23″E	Gabbro	LA-ICPMS	182 ± 2	Zhao L,2019 [55]
31	XLJG-011	Xulaojiugou	47°17’14″N	128°25’40″E	Monzogranite	LA-ICPMS	181 ± 1	Hu X L,2014 [57]
32	XLJG-061	Xulaojiugou	47°17’46″N	128°26’19″E	Monzogranite	LA-ICPMS	180 ± 1	Hu X L,2014 [56]
33	H15-12-3	Shenshuzhen	46°56’19″N	128°27’59″E	Dolerite	LA-ICPMS	187±2	Ge M H,2020(a) [25]
34	DKDA5	Daanhe	46°58’13″N	128°28’40″E	Pyroxene Diorite	LA-ICPMS	195 ± 1	Tan H Y,2012 [54]
35	PM06TC59	Tieli	46°44’35″N	128°29’40″E	Granodiorite	LAICP-MS	183 ±1	Zhen B,2016 [56]
36	LM-0101	Luming	47°22’08″N	128°31’54″E	Monzogranite	LA-ICPMS	181 ± 2	Hu X L,2014 [57]
37	DKL3	Luming	47°22’22″N	128°32’37″E	Alkalic feldspar granite	LA-ICPMS	187±2	Tan H Y,2012 [54]
38	DKL1	Luming	47°22’21″N	128°32’39″E	Alkalic feldspar granite	LA-ICPMS	192±3	Tan H Y,2012 [54]
39	TWL2	Luming	47°22’07″N	128°33’00″E	Monzogranite	LA-ICPMS	187±1	Tan H Y,2012 [54]
40	CHY-2	Cuihongshan	48°50’37″N	128°33’19″E	Quartz monzonite	LA-ICPMS	197 ± 3	Fei X H,2018 [58]
41	LM2	Luming	47°19’25″N	128°34’20″E	Monzogranite	LA-ICPMS	187 ± 2	Cheng G H,2015 [59]
42	PM03TC27	Tieli	46°48’51″N	128°34’33″E	Syenogranite	LA-ICPMS	230 ± 1	Zhen B,2016 [56]
43	LM1	Luming	47°22’02″N	128°36’34″E	Granite -porphyry	LA-ICPMS	183 ± 2	Cheng G H,2015 [59]
44	H15-14-1	Shenshuzhen East	46°57’51″N	128°42’17″E	Monzogranite	LA-ICPMS	191 ± 1	Ge M H,2018 [11]
45	TWC2	Cuihongshan	48°29’05″N	128°44’16″E	Alkalic feldspar granite	LA-ICPMS	182 ± 2	Tan H Y,2013 [60]
46	CHYX-2	Cuihongshan	49°05’45″N	128°46’39″E	Monzogranite	LA-ICPMS	197 ± 1	Fei X H,2018 [58]
47	Jan-73	Tuanjie	46°43’00″N	128°47’14″E	Monzogranite	LA-ICPMS	201 ± 3	Wu F Y,2011 [14]
48	PM03TC06	Tieli	46°47’04″N	128°47’38″E	Monzogranite	LA-ICPMS	197 ± 1	Zhen B,2016 [56]
49	Feb-67	Milin	46°31’05″N	128°49’51″E	Alkalic feldspar granite	K-Ar	197 ± 2	Wu F Y,2011 [14]
50	Jan-66	Langxiang	46°55’42″N	128°53’15″E	Granodiorite	LA-ICPMS	200 ± 2	Wu F Y,2011 [14]
51	DKH12	Huojihe	48°30’31″N	128°56’49″E	Granodiorite	LA-ICPMS	194 ± 1	Tan H Y,2012 [54]
52	ZK0728-2	Huojihe	48°30’55″N	128°57’20″E	Monzonitic Granite Porphyry	LA-ICP-MS	182 ± 1	Ge M H,2016 [61]
53	TWH2	Huojihe	48°30’55″N	128°57’23″E	Granodiorite	LA-ICPMS	181± 2	Tan H Y,2012 [54]
54	00SW225	Hongqi	47°41’50″N	128°58’00″E	Granodiorite	SHRIMP	198 ± 4	Wu F Y,2011 [14]
55	DKD1	Daxilin	47°33’03″N	129°02’16″E	Monzogranite	LA-ICPMS	187 ± 1	Tan H Y,2012 [54]
56	DY0385-1	Chaoxiantun	47°38’48″N	129°02’55″E	alkalic feldspar granite	LA-ICPMS	176 ± 2	Wu F Y,2011 [14]
57	TWS6	Xiaoxilin	47°23’28″N	129°03’53″E	Porphyritic granite	LA-ICPMS	194 ± 1	Tan H Y,2012 [54]
58	H15-16-1	Southeast of langxiangzhen	47°02’49″N	129°06’19″E	Monzogranite	LA-ICPMS	195 ± 2	Ge M H,2018 [11]
59	HTW1-1	Xincun	48°32’24″N	129°12’51″E	Gabbro	LA-ICPMS	185 ± 1	Zhao L,2019 [55]
60	P40-1	Taiqing	47°35’28″N	129°14’23″E	Monzonite	LA-ICPMS	210 ± 2	Wu F Y,2011 [14]
61	DY0380-1	Xiaoxilin	47°27’22″N	129°14’40″E	Granodiorite	LA-ICPMS	200 ± 3	Wu F Y,2011 [14]
62	ECH58-57	Cuihongshan	49°19’54″N	129°15’13″E	Porphyritic granite	LA-ICPMS	194 ± 3	Fei X H,2018 [58]
63	1015–1	Xiaoxilin	47°29’09″N	129°17’56″E	Monzogranite	LAICP-MS	212 ± 2	Liu J F,2008 [30]
64	00SW231	Dafeng	47°24’15″N	129°24’36″E	Monzogranite	SHRIMP	201 ± 4	Wu F Y,2011 [14]
65	HTW6-1	Shuguang	48°29’35″N	129°25’21″E	Gabbro	LA-ICPMS	186 ± 2	Zhao L,2019 [55]
66	P31-4	Fengmao-helin	47°20’54″N	129°36’22″E	Monzogranite	LAICP-MS	211 ± 1	Liu J F,2008 [30]
67	P24-4	Fengmao-helin	47°24’53″N	129°40’57″E	Granite -porphyry	LAICP-MS	210 ± 2	Liu J F,2008 [30]
68	1007–1	Fengmao-helin	47°24’56″N	129°43’28″E	Monzogranite	LAICP-MS	264 ± 1	Liu J F,2008 [30]
69	Feb-80	Qingshuicun	48°16’28″N	129°47’03″E	Alkalic feldspar granite	LA-ICPMS	222 ± 5	Sun D Y,2004 [62]
70	1009–1	Fengmao-helin	47°34’53″N	129°47’18″E	Monzogranite	LAICP-MS	261 ± 1	Liu J F,2008 [30]
71	LZS-1	Lianzhushan	48°28′52″N	129°55’10″E	Monzogranite	LAICP-MS	244 ± 1	Ren L,2017 [63]
72	PDS7-1	Pingdingshan	48°23’34″N	129°55’36″E	Monzogranite	LA-ICPMS	250 ± 3	Bao Z Y,2014 [64]
73	LZS-2	Lianzhushan	48°28′34″N	129°55’40″E	Quartz-diorite	LAICP-MS	215 ± 1	Ren L,2017 [63]
74	P10-2	Fengmao-helin	47°22’51″N	129°59’27″E	Granodiorite	LAICP-MS	244 ± 2	Liu J F,2008 [30]
The south side of Jiamusi-Yilan fault								
75	DY103-2	Liangjiashan	44°26’01″N	126°46’30″E	Alkalic feldspar granite	LAICP-MS	191 ± 2	Wu F Y,2011 [14]
76	DY104-2	Shulan	44°26’18″N	126°53’13″E	Granodiorite	LAICP-MS	190±2	Wu F Y,2011 [14]
77	MG-7	Tianqiaogang	43°50’54″N	126°58’50″E	Syenogranite	SHRIMP	182 ± 3	Sun D Y,2004 [62]
78	15	Laoyeling	43°57′00″N	127°18′02″E	Alkalic feldspar granite	LA-ICPMS	185 ± 2	Sun X,2016 [65]
79	11JJH4-1	Xinzhan	43°59’42″N	127°20’48″E	Gabbrodiorite	LA-ICPMS	256 ± 2	Wang Z J,2016 [66]
80	13JH3-1	Tuding	43°59’42″N	127°20’48″E	Gabbro	LA-ICPMS	252 ± 1	Wang Z J,2016 [65]
81	RT-04	Shenxiandong	44°20’32″N	127°22’45″E	Monzogranite	SHRIMP	180 ± 2	Zhu Y,2017 [66]
82	7	Shangyingbei	44°11′53″N	127°23′11″E	Alkalic feldspar granite	LA-ICPMS	179 ± 3	Sun X,2016 [64]
83	J005	Lafashan	43°48′34″N	127°24′11″E	Alkalic feldspar granite	LA-ICPMS	191 ± 2	Sun X,2016 [64]
84	DP17TW1	Baishila	44°10’40″N	127°32’25″E	Granodiorite	LAICP-MS	224 ± 1	Ao G,2016 [67]
85	DP13TW1	Baishila	44°17’45″N	127°35’21″E	Granite	LAICP-MS	183 ± 1	Ao G,2016 [67]
86	1074TW	Zhangjiawan	44°30’18″N	127°35’47″E	Quartz-diorite	LAICP-MS	171 ± 2	Ren Y J,2019 [23]
87	3082TW	Zhangjiawan	44°31’01″N	127°37’54″E	Syenogranite	LAICP-MS	166 ± 2	Ren Y J,2019 [23]
88	TW7265	Baishila	44°19’56″N	127°40’08″E	Quartz-diorite	LAICP-MS	200 ± 3	Ao G,2016 [67]
89	TW6252	Baishila	44°16’36″N	127°41’34″E	Monzogranite	LAICP-MS	200 ± 1	Ao G,2016 [67]
90	DP12TW9	Baishila	44°04’46″N	127°42’56″E	Granodiorite	LAICP-MS	199 ± 1	Ao G,2016 [67]
91	Jan-28	Sandaohe	43°53’37″N	127°46’13″E	Syenogranite	LA-ICPMS	216 ± 3	Sun D Y,2004 [61]
92	97SW001	WUujimi	45°12’14″N	127°48’13″E	Syenogranite	SHRIMP	180 ± 3	Wu F Y,2011 [14]
93	TW3049	Baishila	44°18’19″N	127°53’32″E	Monzogranite	LAICP-MS	202 ± 2	Ao G,2016 [67]
94	TW6098	Baishila	44°09’52″N	127°56’43″E	Granodiorite	LAICP-MS	200 ± 2	Ao G,2016 [67]
95	DP16TW7	Baishila	44°03’00″N	127°57’32″E	Monzogranite	LAICP-MS	230 ± 1	Ao G,2016 [67]
96	WH	Weihe	44°37′46″N	128°18’24″E	Granite	LAICP-MS	186 ± 2	Geng W,2015 [68]
97	H15-69-1	Qingyunshan	45°07’58″N	128°25’21″E	Syenogranite	LA-ICPMS	189 ± 1	Ge M H,2017 [10]
98	H15-70-1	Qingyunshan	45°07’58″N	128°25’22″E	Syenogranite	LA-ICPMS	200 ± 1	Ge M H,2017 [10]
99	D5343	Pingdingshan	46°30’10″N	128°25’34″E	Alkalic feldspar granite	LA-ICPMS	189±3	Yin Z G,2021 [22]
100	H15-71-1	Chaoyangtun	45°04’35″N	128°30’00″E	Monzogranite	LA-ICPMS	198 ± 1	Ge M H,2017 [10]
101	97SW101	Yanshou	45°47’26″N	128°30’13″E	Syenogranite	SHRIMP	199 ± 5	Wu F Y,2011 [14]
102	98SW103	Yanshou	45°47’51″N	128°30’21″E	Granodiorite	SHRIMP	191 ± 4	Wu F Y,2011 [14]
103	11HNA11-1	Huangqigou	43°49’39″N	128°33’16″E	Granodiorite	LA-ICPMS	252 ± 2	Yu J J,2013 [69]
104	11HNA4-1	Huangqigou	44°06’16″N	128°43’19″E	Monzogranite	LA-ICPMS	255 ± 2	Yu J J,2013 [69]
105	TW-02	Shuguang Forest Farm	45°40’34″N	129°17’05″E	Monzogranite	LAICP-MS	187±1.6	this article
106	TW-04	Shuguang Forest Farm	45°42’30″N	129°22’19″E	Diorite	LAICP-MS	197±2.9	this article
107	16GW126	Hailin	44°38’27″N	129°22’39″E	Granodiorite	LA-ICPMS	219±1	Zhao D,2018 [1]
108	16GW124	Hailin	44°38’27″N	129°22’39″E	Diorite	LA-ICPMS	221±1	Zhao D,2018 [1]
109	16GW121	Hailin	44°39’28″N	129°22’53″E	Monzogranite	LA-ICPMS	217±1	Zhao D,2018 [1]
110	TW-05	Shuguang Forest Farm	45°41’37″N	129°24’07″E	Gabbro	LAICP-MS	193±2.5	this article
111	TW-03	Shuguang Forest Farm	45°46’17″N	129°24’27″E	Granodiorite	LAICP-MS	194±2.8	this article
112	11HNA1-1	Huangqigou	44°23’02″N	129°26’19″E	Quartz-diorite	LA-ICPMS	256 ± 1	Yu J J,2013 [69]
113	TW-01	Shuguang Forest Farm	45°37’23″N	129°27’33″E	Syenogranite	LAICP-MS	192±2.4	this article
114	14GW005	Zhushan	46°25’43″N	129°29’31″E	Meta-gabbro	LA-ICP-MS	259 ± 3	Dong Y,2018 [12]
115	16GW114	Hailin	44°40’29″N	129°34’23″E	Granodiorite	LA-ICPMS	215±1	Zhao D,2018 [1]
116	16GW110	Hailin	44°40’29″N	129°34’23″E	Diorite	LA-ICPMS	221±1	Zhao D,2018 [1]
117	18HL-07-1	Yilan	46°25’35″N	129°34’55″E	Monzogranite	LA-ICPMS	257 ± 3	Ge M H,2020(b) [26]
118	D001	Yilan	46°17’52″N	129°36’59″E	Meta-gabbro	LA-ICPMS	256 ± 3	Zhu Y,2017 [66]
119	14GW079	Hexing	45°42’54″N	129°59’52″E	Syenogranite	LA-ICPMS	246 ± 2	Yang H,2017 [50]
120	14GW083	Dongfanghong	45°41’49″N	130°00’12″E	Granodiorite	LA-ICPMS	253 ± 3	Yang H,2017 [50]
121	14GW107	Damadang	45°25’10″N	130°06’59″E	Granodiorite	LA-ICPMS	258 ± 2	Yang H,2017 [50]
122	14GW074	Shengchan	45°51’51″N	130°07’38″E	Monzogranite	LA-ICPMS	258 ± 2	Yang H,2017 [50]
123	14GW088	Tuchengzi	45°41’33″N	130°1’22″E	Quartz-diorite	LA-ICPMS	250 ± 2	Yang H,2017 [50]
124	14GW068	Jinfeng	45°51’22″N	130°15’23″E	Syenogranite	LA-ICPMS	254 ± 2	Yang H,2017 [50]
125	14GW067	Fuxing	45°51’55″N	130°17’12″E	Granodiorite	LA-ICPMS	256 ± 2	Yang H,2017 [50]
126	15GW073	Mingyi	46°34’21″N	130°20’21″E	Granodiorite	LA-ICP-MS	278 ± 2	Dong Y,2018 [12]
127	15GW248	Hengtoushan	46°54’35″N	130°30’12″E	Monzogranite	LA-ICP-MS	267 ± 3	Dong Y,2018 [12]
128	15GW265	Qingbei	46°45’20″N	130°30’45″E	Monzogranite	LA-ICP-MS	266 ± 2	Dong Y,2018 [12]
129	15GW075	Mengjiagang	46°21’19″N	130°32’24″E	Monzogranite	LA-ICP-MS	272 ± 2	Dong Y,2018 [12]
130	14GW364	Daerlong	45°30’34″N	130°36’49″E	Granodiorite	LA-ICPMS	275 ± 2	Yang H,2017 [50]
131	15GW235	Tuoyaozi	46°03’52″N	130°43’36″E	Syenogranite	LA-ICP-MS	276 ± 3	Dong Y,2018 [12]

### Petrogenesis

#### Petrogenesis and magmatic evolution of the granites

**Fig 9:**
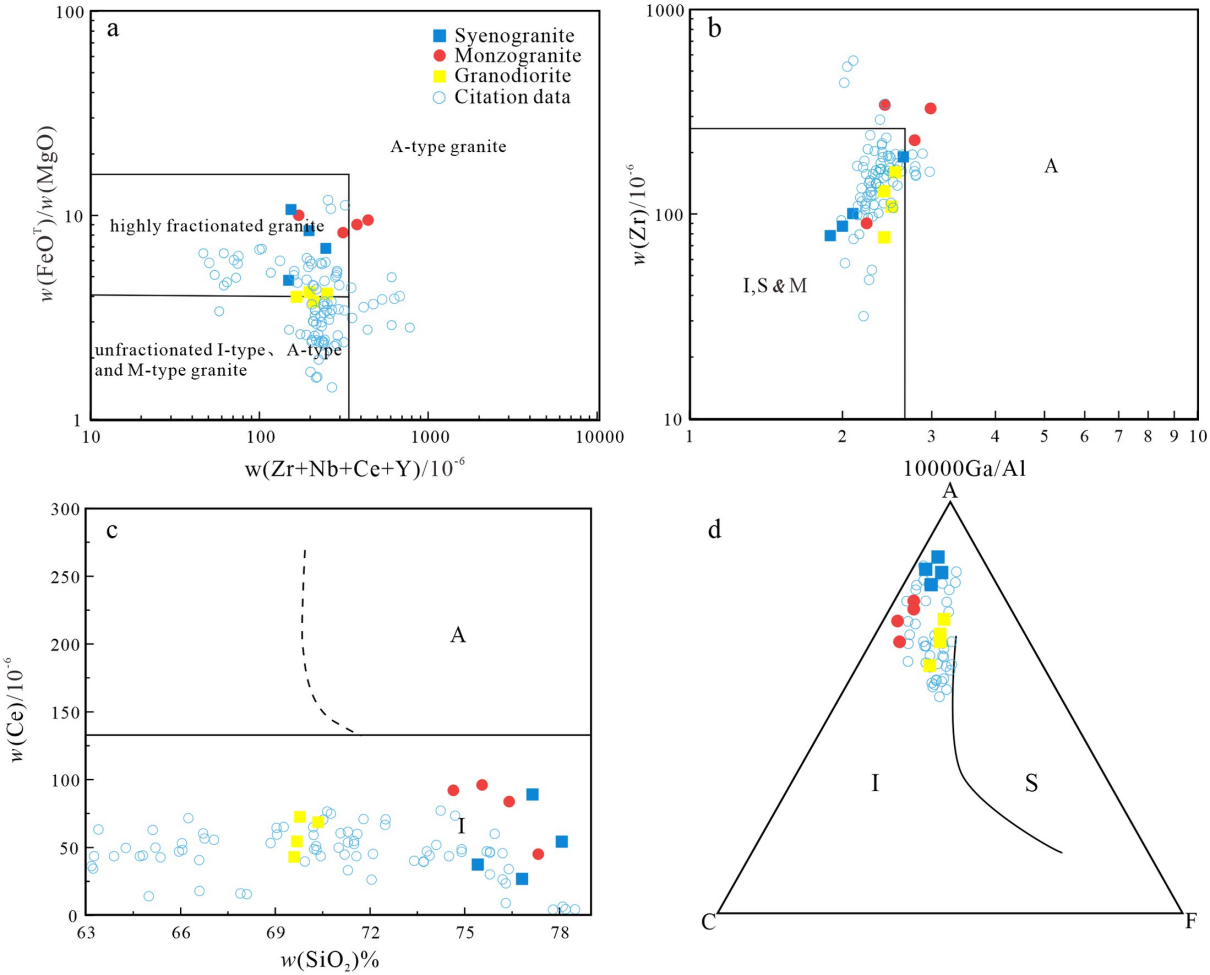
(a) FeO^T^/MgO-*w*(Zr+Nb+Ce+Y)diagram (Base map is cited from [74]); (b) Zr-Ga/Al diagram (Base map is cited from [74]); (c) Ce-SiO_2_ diagram (Base map is cited from [75]); (d) AFC diagram of granite in the study area (Base map is cited from [76]) (The reference data come from [1, 22, 26, 31, 47, 48]).

The types of granitoids include syenogranite, monzogranite, and granodiorite. They consist mainly of quartz, plagioclase, K-feldspar, and amphibole, along with accessory apatite, zircon, magnetite, and titanite. The granitoids do not contain minerals characteristic of typical S-type granites, such as muscovite, garnet, and cordierite [71]. The samples are metaluminous to weakly peraluminous, with A/CNK < 1.1. These geochemical characteristics are distinct from those of S-type granites [72, 73]. All samples plot in the I-type granite field in an AFM diagram (Fig 9d). However, highly fractionated I- and A-type granites can exhibit similar mineralogical and geochemical features [72].

Alkaline mafic minerals (riebeckite—arfvedsonite and aegirine—augite) and Fe-rich olivine (fayalite) are usually important mineralogical indicators of A-type granites [19, 26]. The studied granites contain alkali feldspar, quartz, plagioclase, and minor biotite and amphibole, but do not include alkaline mafic minerals. The mineralogy is typical of highly fractionated I-type granites. The granite samples plot in the I-type granite field in FeO^T^/MgO–(Zr + Nb + Ce + Y), Zr–10000Ga/Al, and Ce–SiO_2_ diagrams (Fig 9a–9c). Furthermore, the low zircon saturation temperature (T_zr_ = ~779°C) of the samples differs from those of A-type granites (>800°C) (Table 2), but is consistent with those of highly fractionated I-type granites.

I-type granites can form by: (1) fractional crystallization of mantle-derived mafic magmas [77]; (2) partial melting of lower crustal material [78]; or (3) mixing of mantle- and crustal-derived magmasl [79]. The studied granitoids are characterized by high silica and alkali contents, and low MgO and CaO contents. They are metaluminous to peraluminous, high-K calc-alkaline rocks, with relatively low Mg^#^ values. These features indicate the granitic magmas were derived from the crust.

**Fig 10:**
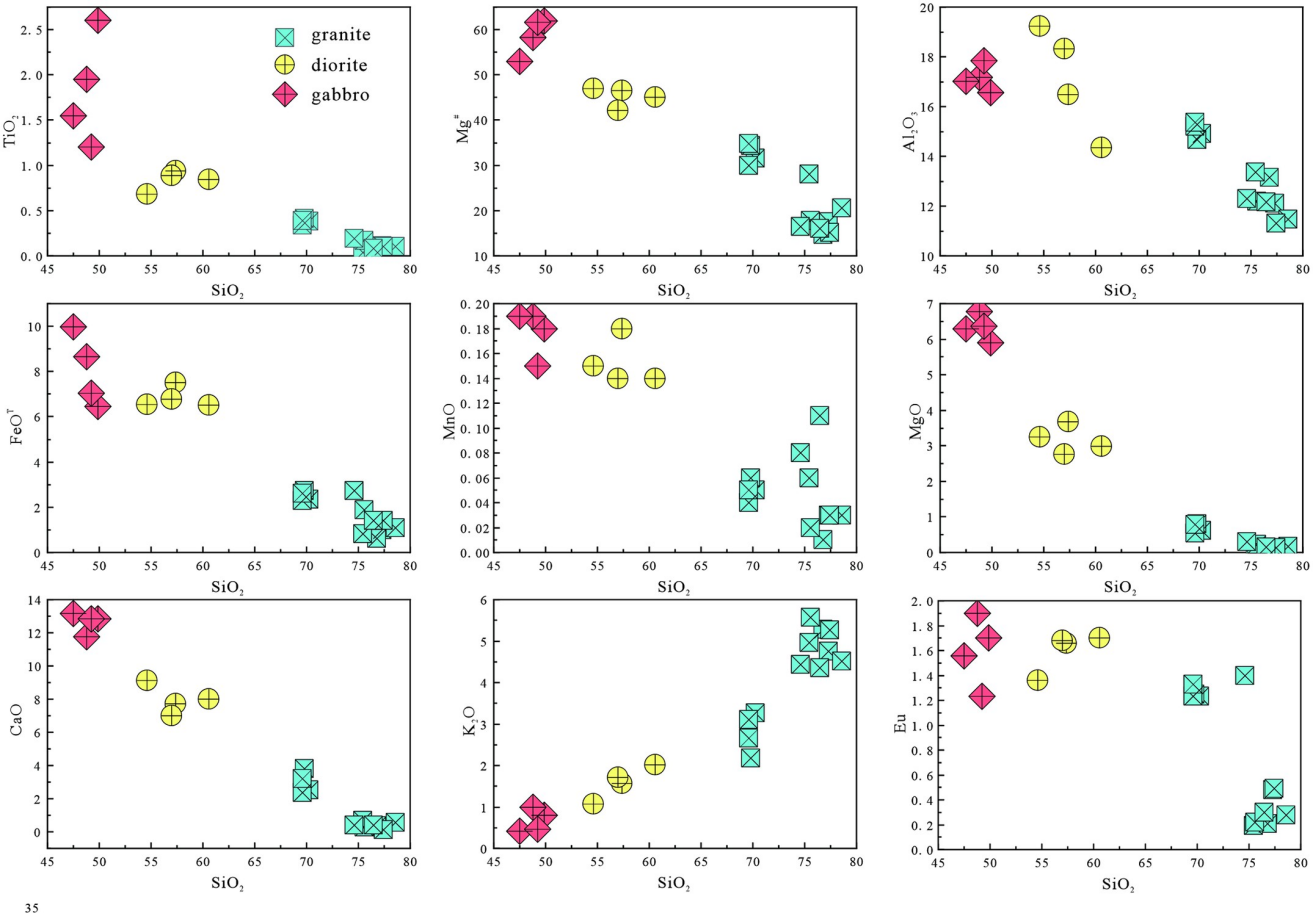
Harker variation diagrams for the intrusive rocks.

The granites have relatively high SiO_2_ contents and A/CNK values, and negative anomalies in Nb, Ta, P, Eu, and Ti, indicating the magmas experienced fractional crystallization [80, 81]. In Harker diagrams (Fig 10), TiO_2_, Al_2_O_3_, MnO, CaO, and Mg^#^ decrease with increasing SiO_2_, whereas K_2_O increases. These features are consistent with fractional crystallization of biotite, plagioclase, amphibole, titanite, and apatite. The Eu anomalies are due to fractional crystallization of K-feldspar and plagioclase. The P depletion is due to fractional crystallization of apatite, and the Ti depletion was caused by fractional crystallization of minerals such as ilmenite and titanite. These inferred fractionating phases are consistent with the petrography.

#### Petrogenesis and magmatic evolution of the diorites

**Fig 11:**
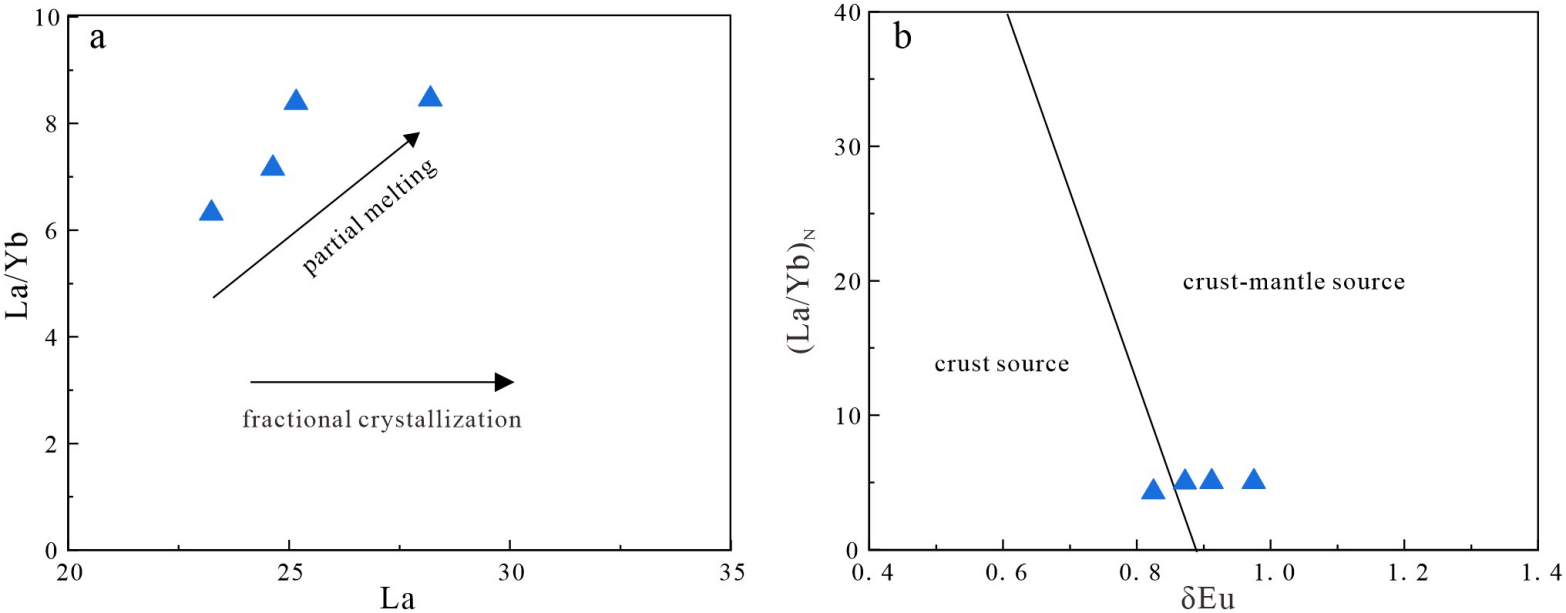
(a) La/Yb-La diagram (Base map is cited from [87]); (b) (La/Yb)_N_-δEu diagram (Base map is cited from [87]).

**Fig 12:**
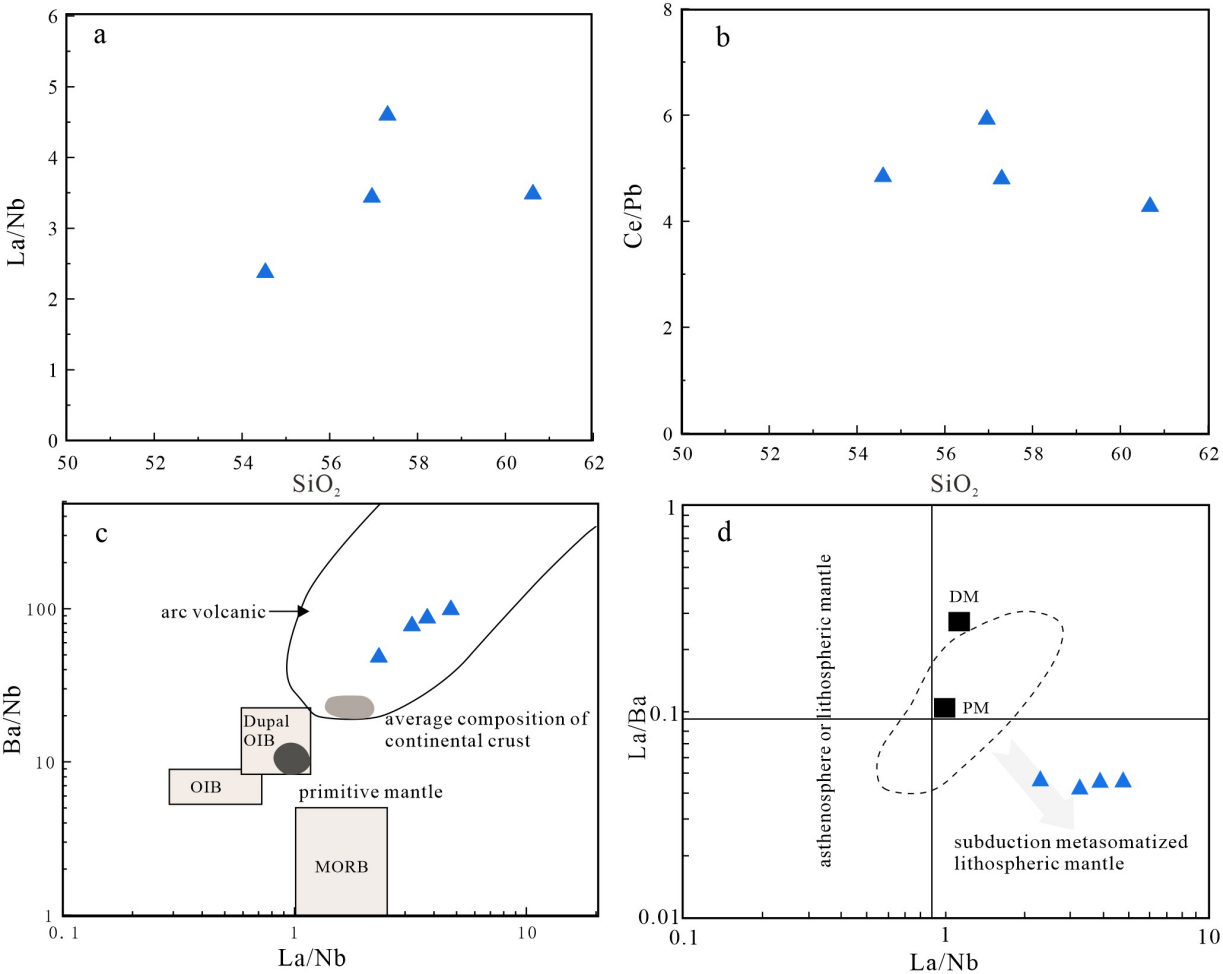
(a) La/Nb-SiO_2_ diagram (Base map is cited from [87]); (b) Ce/Pb-SiO_2_ diagram (Base map is cited from [87]); (c) Ba/Nb-La/Nb diagram (Base map is cited from [91]); (d) La/Ba-La/Nb diagram (Base map is cited from [92]).

Previous studies have proposed three models for the formation of diorites: (1) melting of mafic lower crust [82]; (2) fractional crystallization of mantle-derived magma [83]; and (3) partial melting of mantle metasomatized by subduction-related components [84, 85]. The magmas produced by melting of mafic lower crust typically have Mg^#^ values of <40. Diorites formed by fractional crystallization of mantle-derived magmas generally have high Mg^#^ values (>60) and low TiO_2_ contents (<0.5 wt.%) [86]. However, the studied diorites have low Mg^#^ values (42–47) and high TiO_2_ contents (0.70–0.94 wt.%). In Harker diagrams (Fig 10), TiO_2_, FeO^T^, MnO, MgO, and Mg^#^ are negatively correlated with SiO_2_ contents, indicating the diorites underwent fractional crystallization of amphibole and plagioclase. The samples also define a partial melting trend in a La/Yb–La diagram (Fig 11a), which is not consistent with fractional crystallization. Most of the diorite samples do not plot in the crust source field in a (La/Yb)_N_–δEu diagram (Fig 11b), indicating the magmas were not derived from the lower crust. Therefore, the diorites were not derived from the mafic lower crust or by fractional crystallization of mantle-derived magma. The diorites have low SiO_2_ and high MgO contents, and are enriched in transition elements, such as V, Ni, Co, and Cr. The average Ti/Zr ratio is 36.5, which is inconsistent with the Ti/Zr ratio of crust-derived magmas (Ti/Zr < 30). The Nb/Ta ratios are 16.24–19.05, with an average of 17.17, similar to the mantle average of 17.5 [50]. The samples plot in the subduction-metasomatized lithospheric mantle field on a La/Ba–La/Nb diagram (Fig 12d). Therefore, the source of the diorites was mantle that had been metasomatized by subduction components.

**Fig 13:**
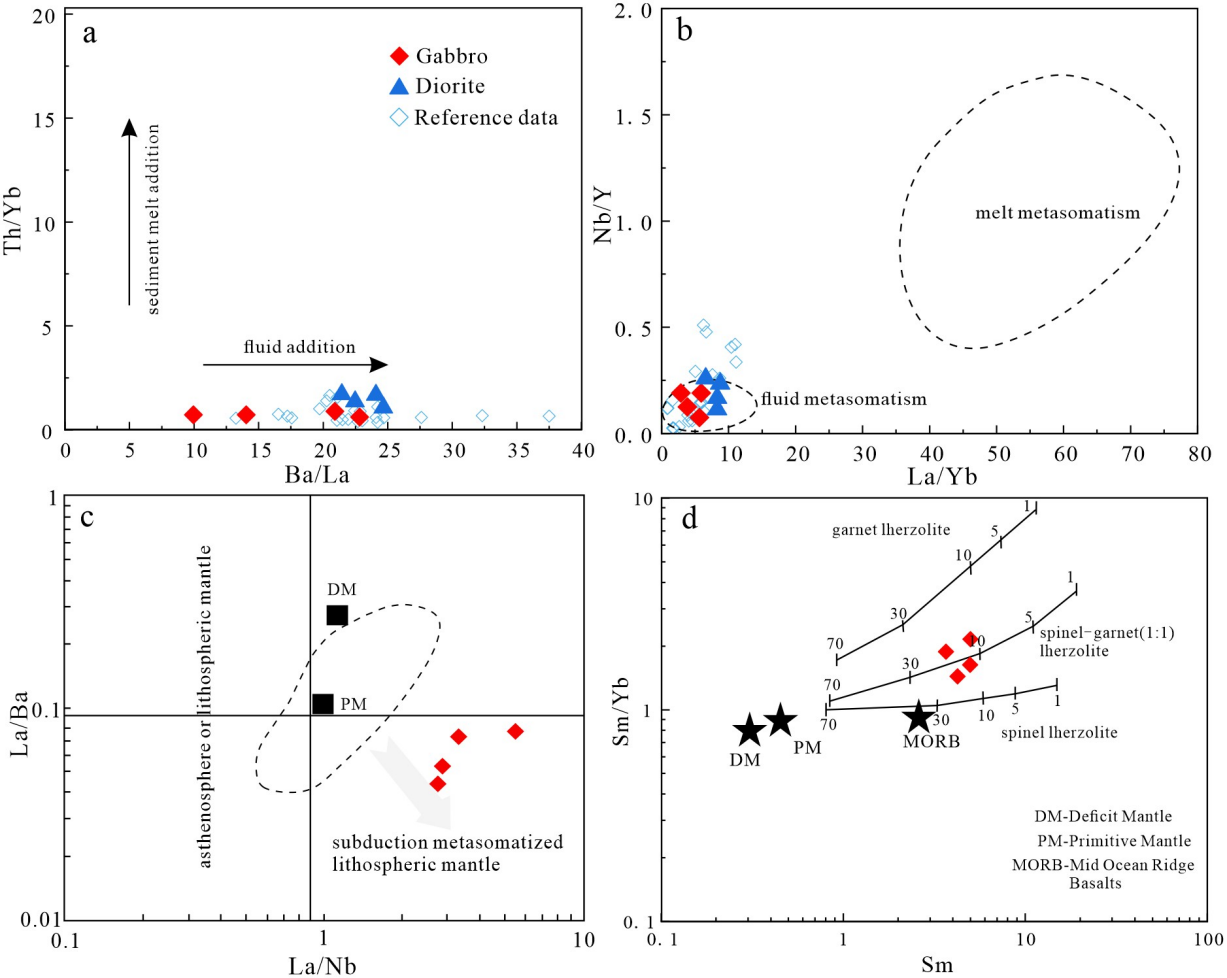
(a) Th/Yb-Ba/La diagram (Base map is cited from [93]); (b) Nb/Y-La/Yb diagram (Base map is cited from [89]); (c) La/Ba-La/Nb diagram (Base map is cited from [92]); (d) Sm/Yb-Sm diagram(Base map is cited from [94]) (The reference data come from [9, 25, 31]).

Mantle-derived magmas can be contaminated by crust during magma ascent. In general, there is a significant linear relationship between Ce/Pb or Nb/La ratios and SiO_2_ contents when diorites experience crustal contamination [88]. However, the studied diorites lack this linear relationship (Fig 12a and 12b). In addition, the diorites have no obvious positive Zr anomalies. In a Ba/Nb–La/Nb diagram (Fig 12c), the samples plot in the arc volcanic field and not between the crust and mantle fields, which precludes the possibility of significant crustal contamination. If the mantle source had been metasomatized by subduction zone melts, it would be relatively enriched in Th and light rare earth elements (LREEs; Th/Yb ≥ 2). However, the Th/Yb ratios of the diorites in the study area are 0.97–1.54, indicating the dioritic magma was not derived from a source that had been metasomatized by sediment-derived melt [89, 90]. In addition, in Th/Yb–Ba/La and Nb/Y–La/Yb diagrams (Fig 13a and 13b) the diorite samples plot in the field of fluid metasomatism, indicating the dioritic magma was derived from a source that had been metasomatized by fluids generated by dehydration of a subducted oceanic plate.

#### Petrogenesis and magmatic evolution of the gabbros

**Fig 14:**
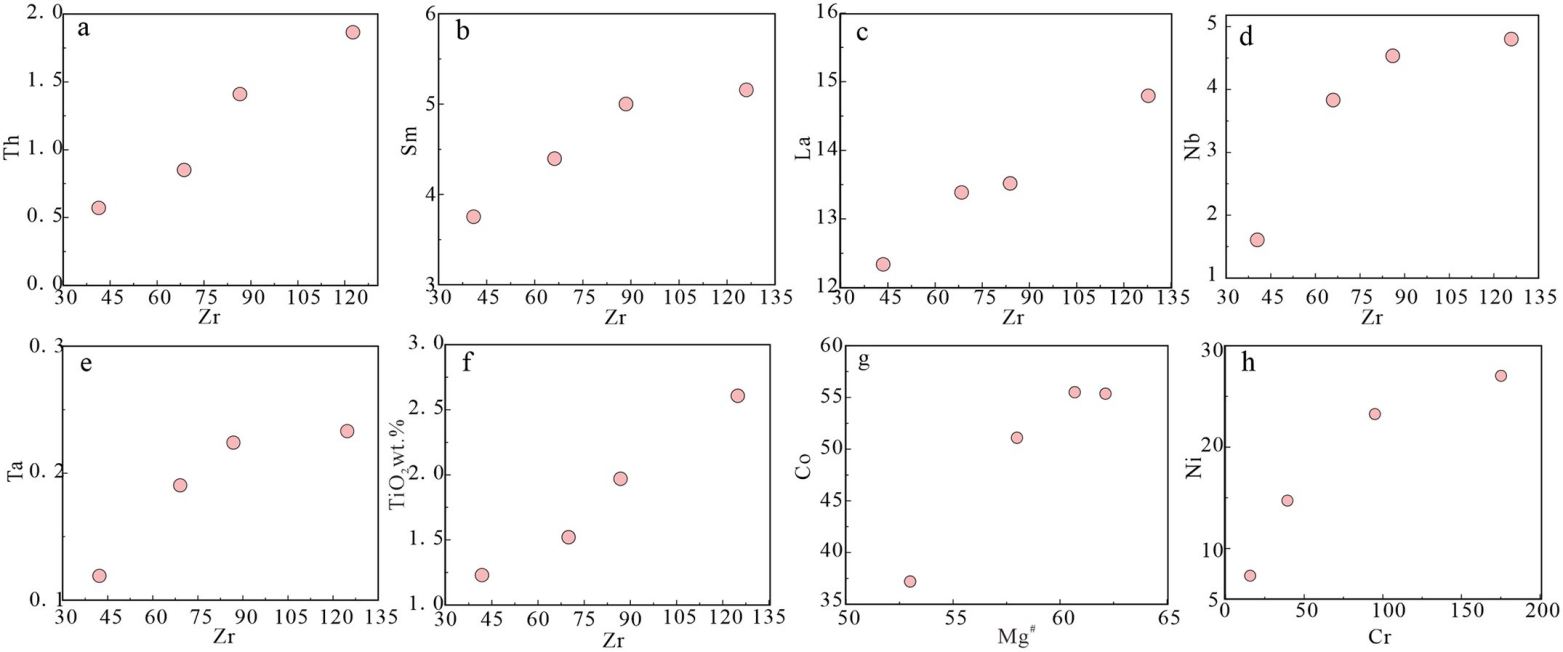
Discrimination diagrams for the gabbro-related elements.

The studied gabbros consist of pyroxene, plagioclase, amphibole, and olivine, and the amphibole is altered, which may have affected the geochemistry. The element Zr is used to assess the mobility of other elements because of its immobile characteristics [95–97]. There is a positive correlation between Zr and Th, Sm, La, Nb, Ti, and Ta contents, indicating these elements were not affected by alteration (Fig 14) [98].

The gabbros have low SiO_2_ contents, high Mg^#^ values, and high TiO_2_ contents, indicating they were derived by partial melting of the mantle [99, 100]. Mantle-derived magmas are susceptible to crustal contamination during magma ascent [101]. The continental crust is strongly depleted in Nb, Ta, and Ti [50, 102]. If the magma undergoes crustal contamination, it will develop island arc-like or crust-like geochemical properties [103]. The studied gabbros are characterized by enrichments in large-ion lithophile elements (LILEs; Ba and K) and depletions in high-field-strength elements (HFSEs; Ta and Ti), indicative of mantle-derived magma contaminated by continental crust. However, other evidence suggests that the mantle-derived magmas were not contaminated by the crust. Despite the relative enrichment of Zr and Hf in the continental crust, Zr in the studied samples does not exhibit obvious positive anomalies in primitive mantle-normalized trace element diagrams (Fig 7d). In addition, compared with the continental crust, the gabbros have lower Th/Ce (0.02–0.06), Th/La (0.04–0.14), and Th/Yb (0.27–0.74) ratios, and higher Nb/Th (2.56–4.45) ratios. These results indicate the mafic magmas might not contaminated by the crust. Therefore, the parental magmas of the gabbros in the Shuguang Forest Farm area were not obviously contaminated by the crust during magma ascent. As such, the negative Ta and Nb anomalies reflect those of the mantle source.

Fractional crystallization also occurs during magma ascent and changes the geochemical composition of the magma. In Harker diagrams (Fig 10), TiO_2,_ FeO^T^, and MgO are correlated negatively with SiO_2_, indicating fractional crystallization of plagioclase, Fe–Ti oxides, and pyroxene. On Co–Mg^#^ and Ni–Cr diagrams (Fig 14), there are positive correlations, indicative of olivine and pyroxene fractional crystallization during formation of the gabbros [104, 105]. The gabbros do not exhibit an obvious negative Eu anomaly in chondrite-normalized REE diagrams (Fig 7c), indicating limited plagioclase fractionation.

The gabbros is enriched in LREEs and LILEs, and depleted in HREEs and HFSEs, and it can be inferred that the gabbroic magma was derived from a mantle source that had been metasomatized by subduction-related fluids or melts [104–109]. Previous studies have shown that fluid derived from a subducted slab is enriched in Ba, Rb, U, and Pb, and depleted in LREEs, Th, and HFSEs (e.g., Zr, Nb, and Ta) [110, 111]. The melts produced by partial melting of subducted sediments are enriched in LREEs, Th, Rb, Ba, U, Sr, and Pb [112]. The gabbroic samples of this study are enriched in Ba, K, and Sr, and depleted in Nb, Ta, and Th (Fig 7d), indicating their source was metasomatized by subduction-related fluids. Source modification by a fluid rather than a sediment-derived melt is consistent with the Th/Yb–Ba/La (Fig 13a) and Nb/Y–La/Yb plots (Fig 13b). In addition, the Th/Yb ratios of the studied gabbros are 0.27–0.74, indicative of the source being modified by subduction-related fluids [89, 90].

The REE contents and ratios can be used to infer the origins and degree of melting of mantle-derived magmas [94, 113]. The Sm/Yb ratio can be used to identify the mineralogy of the mantle source [94]. In La/Ba–La/Nb (Fig 13c) and Sm/Yb-Sm diagrams (Fig 13d), the samples plot in the subduction metasomatized lithospheric mantle field and near the partial melting trend for spinel–garnet (1:1) lherzolite. This indicates the mantle source was spinel–garnet lherzolite, and the degree of partial melting was 10%–20%.

### Tectonic implications

**Fig 15:**
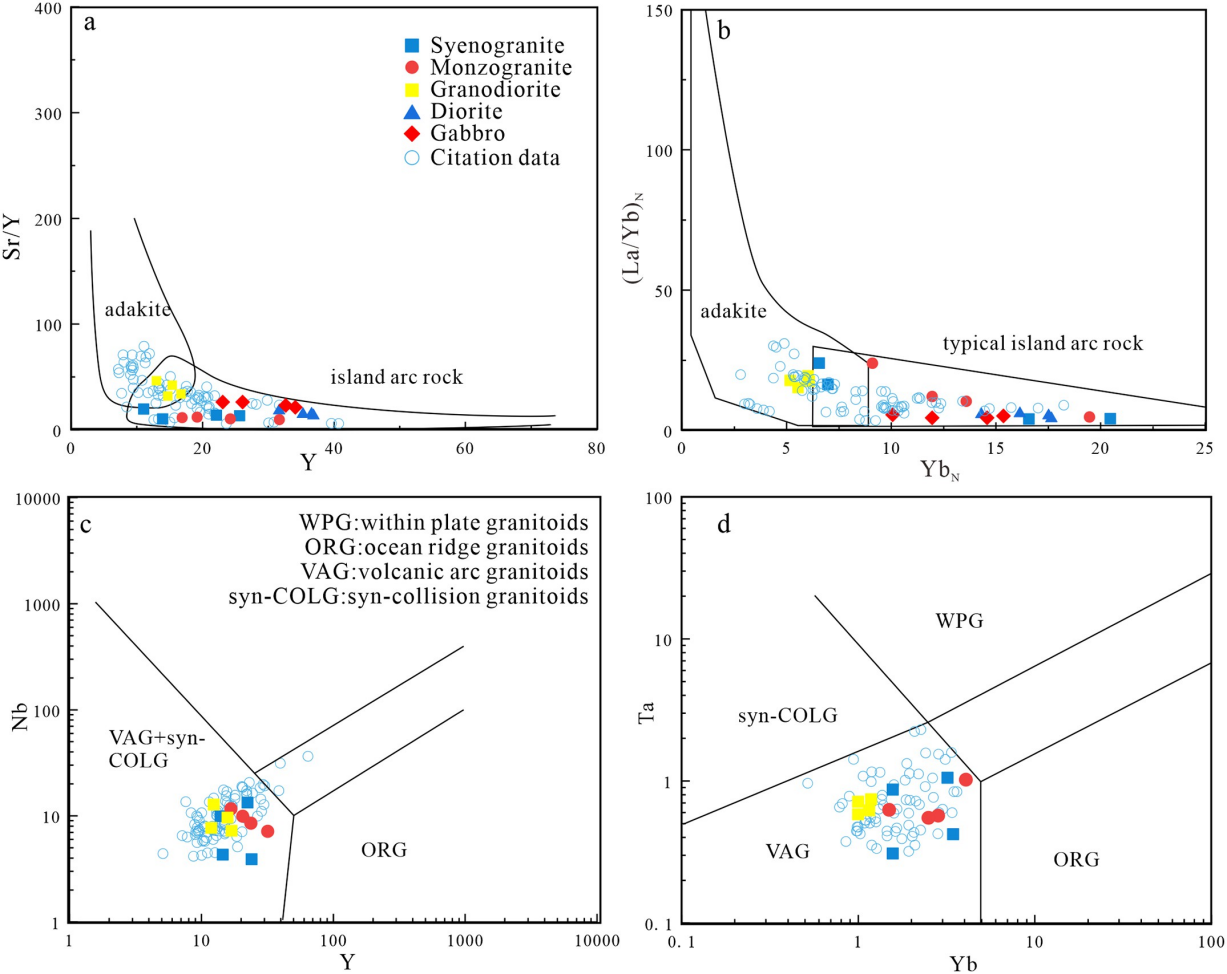
(a) Sr/Y-Y diagram (Base map is cited from [116]); (b) (La/Yb)_N_-Yb_N_ diagram (Base map is cited from [116]); (c) Nb-Y diagram (Base map is cited from [117]); (d) Ta-Yb diagram (Base map is cited from [117]) (The reference data come from [1, 9, 22, 25, 26, 31, 47–49]).

**Fig 16:**
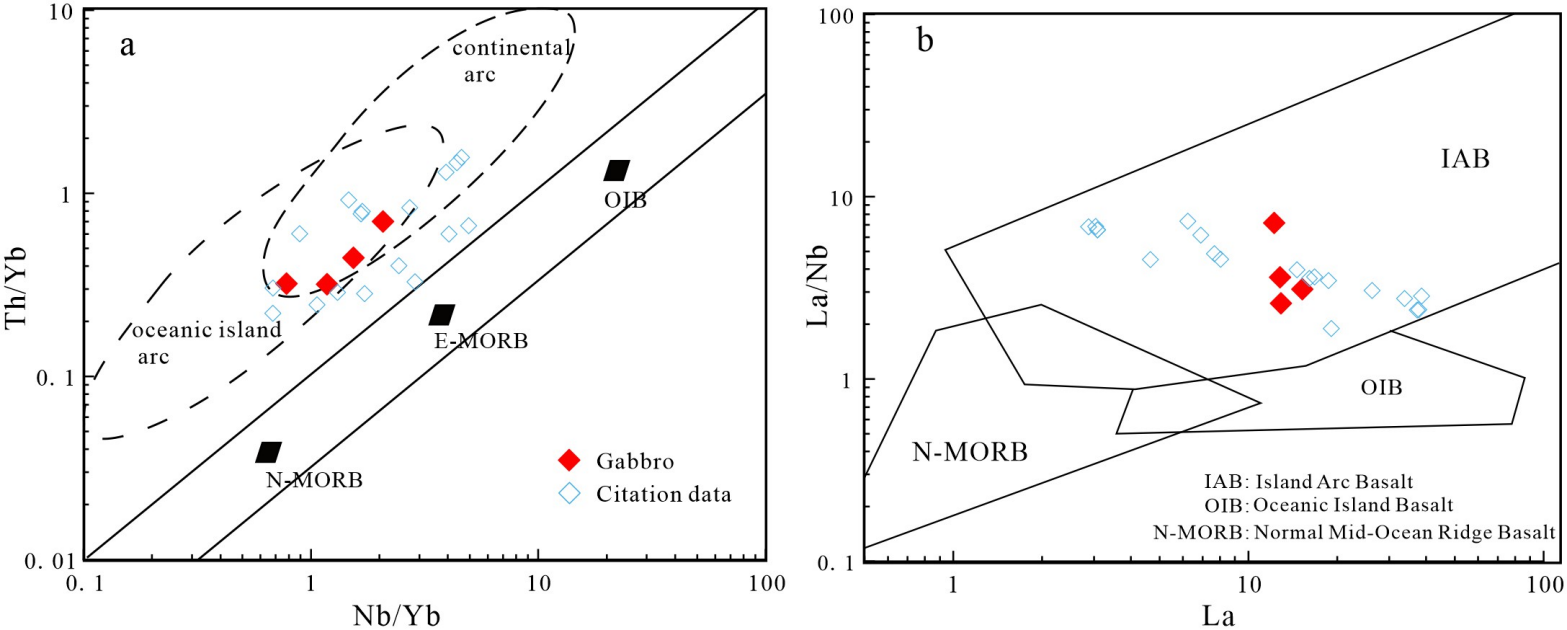
(a) Th/Yb-Nb/Yb diagram (Base map is cited from [96]); (b) La/Nb-La diagram (Base map is cited from [118]) (The reference data comes from [9, 25, 31]).

**Fig 17:**
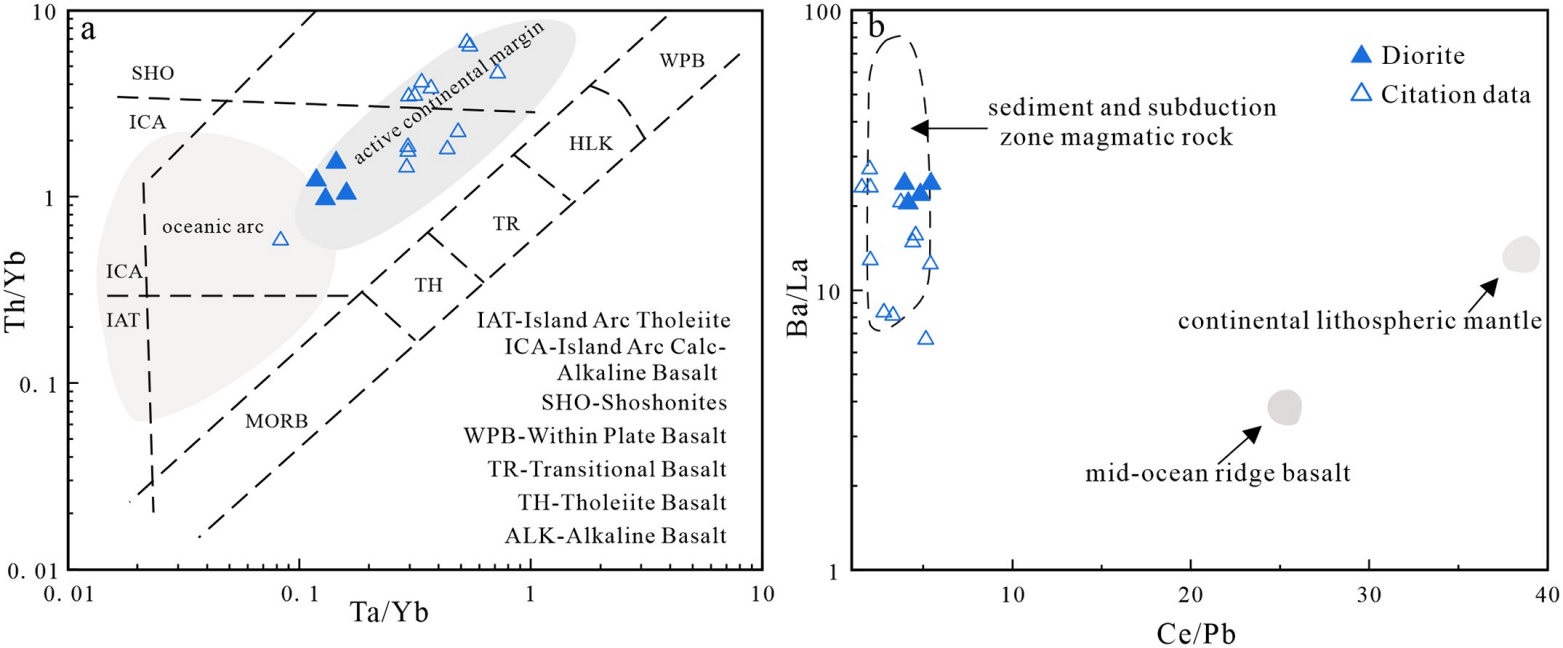
(a) Th/Yb-Ta/Yb diagram (Base map is cited from [119]); (b) Ba/La-Ce/Pb diagram (Base map is cited from [120]) (The reference data come from [1, 47, 48]).

The Lesser Xing’an–Zhangguangcai Range is located between the Jiamusi and Songnen massifs, and contains Late Paleozoic-Mesozoic igneous rocks. Most of these rocks are part of the medium- to high-K calc-alkaline series. They are enriched in LREEs and LILEs, and depleted in HREEs and HFSEs. This suggests they have arc-related characteristics typical of igneous rocks associated with subduction zones [1, 25, 26, 47–49, 114, 115]. Discrimination diagrams for the granites, diorites, and gabbros in the study area show the studied rocks plot in the arc-related pluton field (Fig 15a and 15b). Our samples and other granites in this region almost all plot in the island arc granite field (Fig 15c and 15d). In a Th/Yb–Nb/Yb diagram (Fig 16a), the gabbroic samples exhibit geochemical characteristics related to subduction, and plot in the island arc basalt field in a La–La/Nb diagram (Fig 16b). The studied diorites plot mainly in the active continental margin field in a Ta/Yb–Th/Yb tectonic discrimination diagram (Fig 17a). In a Ce/Pb–Ba/La diagram (Fig 17b), the samples all plot in and near the sediment and subduction zone igneous rock fields. This indicates their formation environment was related to subduction and that their source was modified by sediments.

The Lesser Xing’an—Zhangguangcai Range was located in an active continental margin environment during the Jurassic, and the magma source was modified by subduction-related fluid derived from oceanic lithosphere [1, 9, 121, 122]. Previous studies of Early—Middle Triassic collisional granites from the southern margin of the Xingmeng Orogenic Belt have concluded that the Paleo-Asian Ocean closed in the Early—Middle Triassic. Late Triassic bimodal igneous rocks in the Zhangguangcai Range suggest the region was in an extensional setting during this period, which also indicates the Paleo-Asian Ocean closed before the Late Triassic [3, 31, 123, 124]. Therefore, the tectonic setting in the Early Jurassic was not related to Paleo-Asian Ocean. The ‘Mudanjiang Ocean’ refer to an ancient ocean between the Jiamusi and Songnen—Zhangguangcai Range massifs [14]. The Mudanjiang Ocean may have been the dominant factor in the Mesozoic tectonic evolution of this region. Therefore, the tectonic evolution of the Mudanjiang Ocean is of significance in understanding the tectonic history of these two massifs. The timing and polarity of subduction in the Mudanjiang Ocean are controversial. Liu et al.(2019) determined the age of an ophiolitic melange in northeast China, and concluded that the Mudanjiang Ocean existed from the Late Permian to Middle Jurassic [15]. Based on a study of the Heilongjiang Complex and detrital zircon geochronology, Sun et al.(2018) and Xu et al.(2019) suggested that the Mudanjiang Ocean existed during the Middle Triassic—Early Jurassic [17, 125]. Based on the ages of blueschists and plagioclase amphibolites with ocean island basalts (OIB) and mid ridge island basalts (MORB), Dong Y.(2018a) proposed that the Mudanjiang Ocean existed during the Late Paleozoic—Early Jurassic [12]. Given the temporal and spatial distribution of arc magmatism, as well as its petrogenesis, it is inferred that the subduction polarity in the Mudanjiang Ocean was mainly westward [11, 16] and/or bidirectional [12, 13, 18].

**Fig 18:**
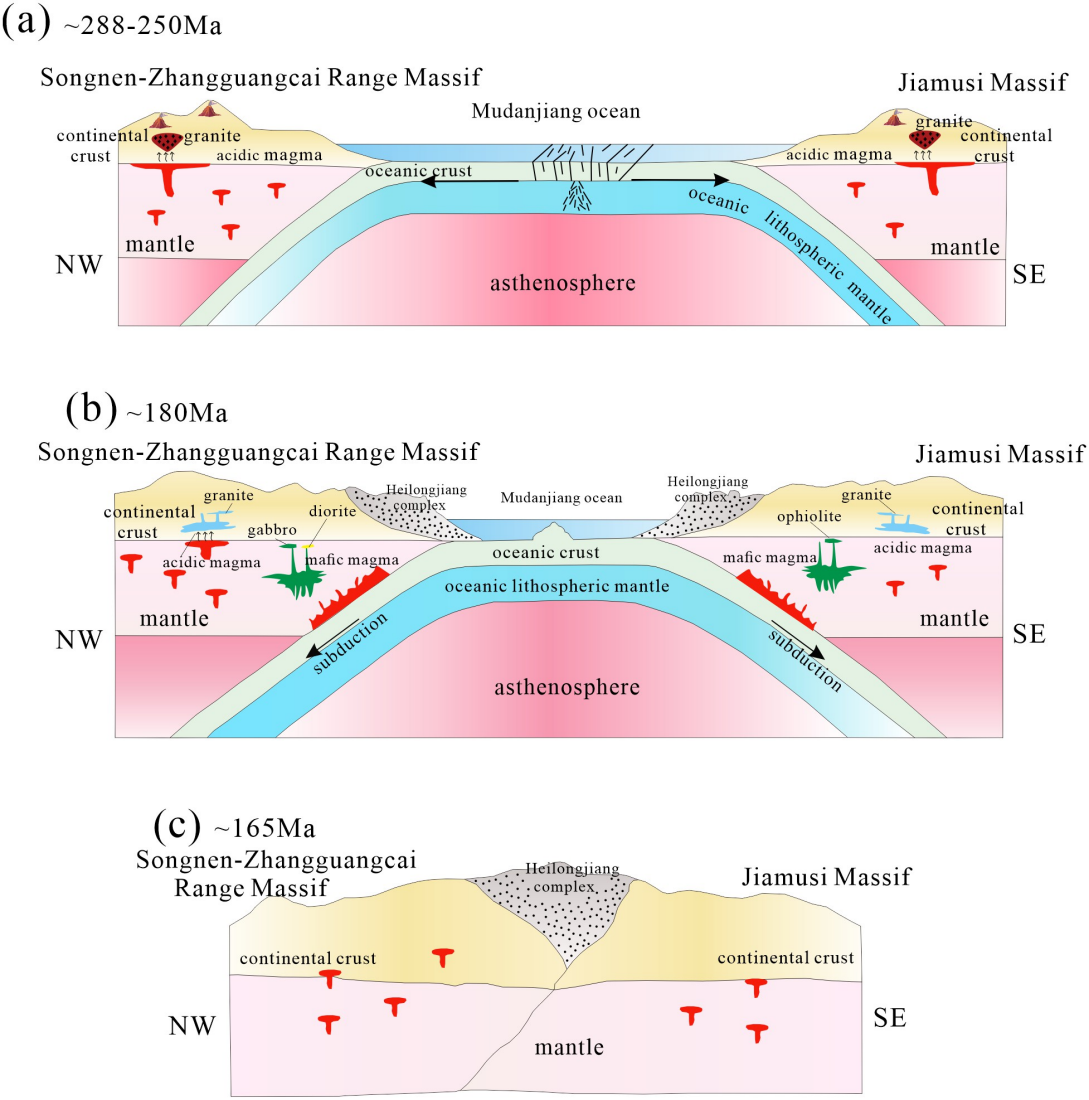
Subduction evolution model of the Mudanjiang ocean(modified from [12]).

Based on the temporal and spatial distribution, petrogenesis, and tectonic setting of igneous rocks in the Lesser Xing’an—Zhangguangcai Range on the eastern margin of the Songnen and Jiamusi massifs, we consider that the Mudanjiang Ocean existed from the Early Permian to Middle Jurassic, and the subduction polarity was bidirectional (Fig 18).

1. During 288–250 Ma, the Mudanjiang Ocean had already opened and was undergoing bidirectional subduction. Ge et al.(2017, 2018) obtained the protolith ages of blueschists in the Early Permian Heilongjiang Complex in Yilan (tholeiitic series = 288 ± 2 Ma; alkaline series = 288 ± 2 Ma) and showed it had OIB-like features [10, 11], indicating the Mudanjiang Ocean between the Jiamusi and Songnen massifs was open by at least the Early Permian. Dong et al.(2018b) proposed that the Early Permian Yilan amphibolites (274 Ma) in the Heilongjiang Complex formed at an active continental margin [126], indicating the Mudanjiang Ocean had opened and was experiencing subduction at this time. The Permian igneous rocks along the western margin of the Jiamusi Massif and eastern margin of the Songnen Massif are bimodal [6, 13, 127], indicating an extensional environment. The N–S-trending Permian granite belt located along the eastern margin of the Songnen—Zhangguangcai Range Massif (Permian arc-related igneous rocks are distributed in the Lesser Xing’an—Zhangguangcai Range, including the Zhushan [12], Hengtoushan [12], and Huangqigou areas [70]), indicates the Mudanjiang Ocean experienced westward subduction. A N–S-trending belt of Permian granite occurs to the east and west of the Jiamusi Massif. However, Permian arc-related igneous rocks in the eastern Jiamusi Massif (302–260 Ma) [128–132] were formed earlier than Permian arc-related igneous rocks in the western Jiamusi Massif (296–246 Ma) [29, 51, 133, 134], indicating the latter were formed by eastward subduction in the Mudanjiang Ocean.
2. Before 180 Ma, the Mudanjiang Ocean continued to experience bidirectional subduction, and the oceanic basin decreased in size. Triassic and Early Jurassic arc-related igneous rocks along the eastern margin of the Songnen—Zhangguangcai Range indicate the Mudanjiang Ocean experienced westward subduction (e.g., in the Taiqing [14], Lianzhushan [63], Tianqiaogang [62], Xingfulinchang [19] and Shuguang Forest Farm areas). There are no Early—Middle Triassic ophiolites or arc igneous rocks along the eastern margin of the Jiamusi Massif, and only Early Triassic (250–246 Ma) I-type granites occur in the Fuxing Forest Farm and Diaoling Town areas of Linkou County along the western margin of the Jiamusi Massif [51], indicating eastward subduction in the Mudanjiang Ocean. The Early Jurassic (197–187 Ma) intrusive rocks in the Shuguang Forest Farm area of the Lesser Xing’an—Zhangguangcai Range formed along an active continental margin, indicating the Mudanjiang Ocean was experiencing subduction at this time. Late Mesozoic—Paleogene intrusive rocks in the Lesser Xing’an—Zhangguangcai Range gradually young from east to west, which also shows that subduction in the Mudanjiang Ocean occurred during the Mesozoic—Paleogene. The Lesser Xing’an—Zhangguangcai Range contains abundant late Paleozoic and Mesozoic igneous rocks that are medium- to high-K calc-alkaline in composition. They are enriched in LREEs and LILEs, and depleted in HREEs and HFSEs, and have arc-like geochemical properties, suggesting the Mudanjiang Ocean was undergoing subduction during this period.
3. Before 160 Ma, the Mudanjiang Ocean continued to undergo bidirectional subduction and finally closed. The metamorphic ages of the Heilongjiang Complex can constrain the timing of closure of the Mudanjiang Ocean. Phengite ^40^Ar/^39^Ar ages (186–165 Ma) of metasedimentary and blueschist-facies rocks in the Heilongjiang Complex are younger than [17, 31, 135], and partly overlap, those of metamorphic zircon and arc-related igneous rocks in the Zhangguangcai Range, suggesting the phengite ^40^Ar/^39^Ar ages record the long-term tectonic exhumation of high-pressure metamorphic rocks due to subduction of the Mudanjiang oceanic lithosphere and collision between the Jiamusi and Songnen massifs. Dong et al. (2018b) reported rutile U–Pb ages (172 ± 5 and 177 ± 11 Ma) for arc-related amphibolites in the Yilan area that are similar to the youngest age of continental arc-related igneous rocks in the Zhangguangcai Range [126]. Yu et al.(2023) suggested the rutile U–Pb ages record local crustal thickening that was caused by the final closure of the Mudanjiang Ocean and the soft collision between the Jiamusi and Songnen massifs [136]. In summary, we conclude that the Mudanjiang Ocean closed during the Middle Jurassic (180–165 Ma).

## Conclusions

1. The ages of intrusive rocks in the study area are 197–187 Ma, including syenogranite (192 ± 2.4 Ma), monzogranite (187 ± 1.6 Ma), granodiorite (194 ± 2.8 Ma), diorite (197 ± 2.9 Ma), and gabbro (193 ± 2.5 Ma). These Early Jurassic ages are consistent with the peak period of magmatism in the Lesser Xing’an—Zhangguangcai Range.
2. The Early Jurassic granites in the study area are highly fractionated I-type granites derived by partial melting of the lower crust, which underwent biotite, plagioclase, amphibole, and titanite fractionation crystallization. The dioritic and gabbroic magmas were derived by partial melting of a mantle source that had been metasomatized by subduction-related fluids and did not undergo obvious crustal contamination. The gabbros underwent fractional crystallization of pyroxene, and the diorites experienced fractional crystallization of amphibole.
3. The Early Jurassic intrusive rocks have arc-like geochemical characteristics, similar to igneous rocks formed in a subduction setting along the eastern margin of the Songnen—Zhangguangcai Range Massif. This indicates they formed in an active continental margin environment, perhaps related to bidirectional subduction in the Mudanjiang Ocean during the Early Jurassic.

## Supporting information

S1 Table Whole-rock geochemical data. (XLSX)
